# Aerosol light absorption alleviates particulate pollution during wintertime haze events

**DOI:** 10.1073/pnas.2402281121

**Published:** 2024-12-23

**Authors:** Jiarui Wu, Naifang Bei, Yuan Wang, Xiaoli Su, Ningning Zhang, Lili Wang, Bo Hu, Qiyuan Wang, Qian Jiang, Chenchong Zhang, Yangfan Liu, Ruonan Wang, Xia Li, Yuxuan Lu, Zirui Liu, Junji Cao, Xuexi Tie, Guohui Li, John Seinfeld

**Affiliations:** ^a^State Key Laboratory of Loess Science, Institute of Earth Environment, Chinese Academy of Sciences, Xi’an 710061, China; ^b^School of Human Settlements and Civil Engineering, Xi’an Jiaotong University, Xi’an, Shaanxi 710049, China; ^c^Department of Earth System Science, Stanford University, Stanford, CA 94304; ^d^State Key Laboratory of Atmospheric Boundary Layer Physics and Atmospheric Chemistry, Institute of Atmospheric Physics, Chinese Academy of Sciences, Beijing 100029, China; ^e^Department of Chemical Engineering, California Institute of Technology, Pasadena, CA 91106

**Keywords:** aerosol light absorption, aerosol–radiation interaction, aerosol–photolysis interaction, PM pollution, numerical simulation

## Abstract

Aerosol light absorption has been widely considered as a contributing factor to the worsening of particulate pollution in large urban areas, primarily through its role in stabilizing the planetary boundary layer (PBL). Here, we report that absorption-dominated aerosol–radiation interaction can decrease near-surface fine particulate matter concentrations ([PM_2.5_]) at a large-scale during wintertime haze events. A “warm bubble” effect by the significant heating rate of absorbing aerosols above the PBL top generates a secondary circulation, enhancing the upward motion (downward motion) and the convergence (divergence) in polluted (relatively clean) areas, with a net effect of lowering near-surface [PM_2.5_]. Furthermore, aerosol absorption of ultraviolet-wave light effectively reduces the photolysis of chemical species, i.e., aerosol–photolysis interaction, hindering ozone formation, reducing atmospheric oxidizing capability, and suppressing secondary aerosol concentrations. Our model assessment reveals that the synergetic two effects decrease near-surface [PM_2.5_] by around 7.4%, so the presence of light-absorbing aerosols can considerably alleviate particulate pollution during wintertime haze events. Such negative feedbacks to the aerosol loading should be considered in weather/climate prediction and health assessment models.

## Significance

Light absorption of anthropogenic aerosol has long been considered as not only an essential contributor to climate warming but also a culprit to deteriorating particulate pollution through suppressing the planetary boundary layer (PBL) development. The research conducted identifies a “warm bubble” effect by light-absorbing aerosols in two mechanisms, one physically and the other chemically based, acting in the same direction and ameliorating wintertime heavily polluted atmospheric conditions. The highlighted aerosol light absorption interactions with atmospheric dynamics and chemistry should be considered in assessment of aerosol radiative forcing and collaborative reduction of air pollutants with escalating global change in the future on a global scale.

Light-absorbing aerosols (e.g., black carbon or BC, brown carbon, and dust) play a unique and vital role in air quality and climate. There is a consensus that BC, as a short-lived climate pollutant, is the second-most critical contributor to climate warming forcing in the present-day atmosphere, behind only CO_2_ (1–3). Moreover, BC has long been considered one of primary causes for particulate matter (PM) pollution during haze events in China, since it is not only an essential component of fine PM (PM_2.5_), but also absorbs solar radiation to increase the atmospheric temperature, modifying the atmospheric stability (4–6). The warming induced by BC, notably in the upper levels of planetary boundary layer (PBL), stabilizes the atmosphere, curbs the development of PBL, and subsequently aggravates PM pollution in China’s major urban areas, referred to as the “dome effect” associated with BC (7).

In contrast to the conventional thinking that light absorption of aerosols deteriorates PM pollution during haze events, absorbing aerosols also have the capacity to elevate the convective available potential energy above PBL, thereby promoting vertical atmospheric motion in the free troposphere and inducing low-level convergence (8). These effects can enhance the summer monsoonal circulation and potentially override the dome effect of absorbing aerosols (9, 10). Severe haze events with high PM_2.5_ mass loading in China are generally characterized by large spatial coverage and long duration (11). For example, a notorious haze event occurred in January 2013 affected ~1.3 million km^2^ and lasted 1 mo (12). Therefore, at a regional scale, the absorption of aerosols has great potential of altering the atmospheric thermodynamics and dynamics.

Absorbing aerosols can also reduce the NO_2_ photolysis rate to affect ozone (O_3_) formation, further reducing atmospheric oxidizing capability (AOC) and secondary aerosol formation (13–15). Note that when forward scattering of ultraviolet (UV) radiation by aerosols dominates over the absorption of UV radiation in the PBL, aerosol can enhance photolysis and facilitate secondary aerosol formation to elevate PM pollution (16). A recent modeling study has demonstrated that a net aerosol effect on photolysis decreases near-surface PM_2.5_ concentration ([PM_2.5_]) by 4.2% on average in the North China Plain (NCP) (17). Nevertheless, the significance of absorption-induced aerosol–radiation interaction (AARI) and aerosol–photolysis interaction (AAPI) on regional air quality is not well understood. Here, we investigate severe PM pollution with varying absorbing aerosols in the NCP, which is recognized as the most polluted area in China. Impacts of AARI and AAPI on PM pollution are assessed based on the model simulations using the Weather Research and Forecast model online coupled with atmospheric chemistry Weather Research Forecasting-Chem (WRF-Chem). Aerosol interactions with radiation, cloud, and photolysis are explicitly simulated in this modeling framework.

## Results and Discussion

To gain a quantitative comprehension of the impact of light-absorbing aerosols on winter PM pollution and the underlying mechanisms, the updated WRF-Chem model is employed here (*SI Appendix*, SI-1). Long-term measurements of single scattering albedo (SSA) during the wintertime from 2010 to 2018 collected at five observation sites in the NCP are used to analyze the association between aerosol optical properties and air pollution.

**Fig. 1.**
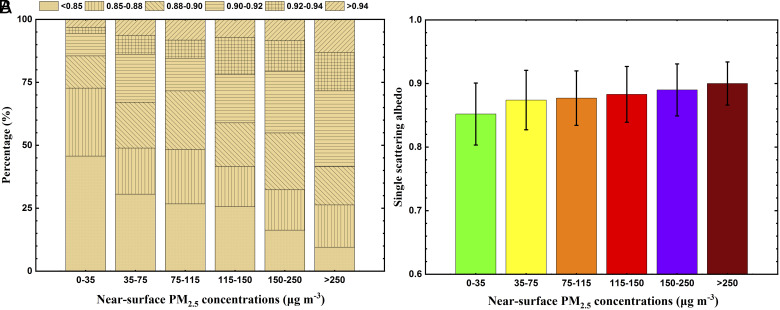
(*A*) The proportion stack bar chart of the observed SSA and (*B*) the average SSA under different air quality grades at five observation sites in the NCP during the wintertime from 2013 to 2018.

The absorbing efficiency of aerosol particles is indicated by SSA, which is further determined by the aerosol composition and mixing state (18). Lower SSA at the visible wavelength indicates stronger absorption of solar radiation by aerosols. ARI and API are not only dependent on the total extinction of radiation by aerosols, i.e., forward/backward scattering ratio (measured by the asymmetry factor), and aerosol optical depth (AOD), but also highly responsive to the SSA. Fig. 1 shows the occurrences of the observed SSA and the average SSA under different air quality grades at five observation sites in the NCP during the wintertime from 2013 to 2018. Moderately strong absorbing aerosols in the atmosphere were observed, with an average SSA of around 0.87. The average SSA shows an increasing trend with deterioration of PM pollution, fluctuating within a narrow range from 0.85 to 0.90, which is caused by an increase in secondary aerosols. As PM_2.5_ levels at the surface fluctuate between 35 and 250 μg m^−3^, the occurrence of SSA lower than 0.92 exceeds 78%, indicating that wintertime aerosols over the NCP possess moderately strong absorbing capability.

To evaluate effects of aerosol absorption on PM pollution, we performed 3-mo WRF-Chem model simulations covering multiple heavy haze episodes that occurred in the NCP with varying absorbing aerosol properties from 05 December 2015 to 06 March 2016 (*SI Appendix*, Fig. S1). A suite of four experiments has been devised (*SI Appendix*, Table S1), including a control scenario (F_BASE_) that incorporates both AARI and AAPI, along with three sensitivity experiments: one that excludes AARI (F_AARI0_), one that excludes AAPI (F_AAPI0_), and one that excludes both AARI and AAPI (F_Abs0_). Simulation results in FBASE are evaluated by available observations (*SI Appendix*, Figs. S2–S19). More technical details can be found in *SI Appendix* for detailed model description, configuration, methodology, and validation. We use an ensemble method to analyze the model sensitivity results (19). For instance, to assess the impact of AARI on PBL height (PBLH) in the NCP during the episode, near-surface [PM_2.5_] in the F_BASE_ are initially divided into 30 bins from 0 to 600 μg m^−3^, each with a 20 μg m^−3^ interval. The PBLH in the same grid cell for both F_BASE_ and F_AARI0_ cases is grouped according to the [PM_2.5_] bins. For each bin, the mean value is subsequently calculated. As depicted in *SI Appendix*, Fig. S20, the event frequency of simulated near-surface PM_2.5_ concentrations in the [PM_2.5_] bins ranging from 20 to 110 μg m^−3^ is more than 10%, and fewer than 1% within the bins above 250 μg m^−3^. The average frequency of severe PM pollution ([PM_2.5_] > 250 μg m^−3^) the occurred in the NCP is approximately 4% during the study episode (*SI Appendix*, Fig. S21).

**Fig. 2.**
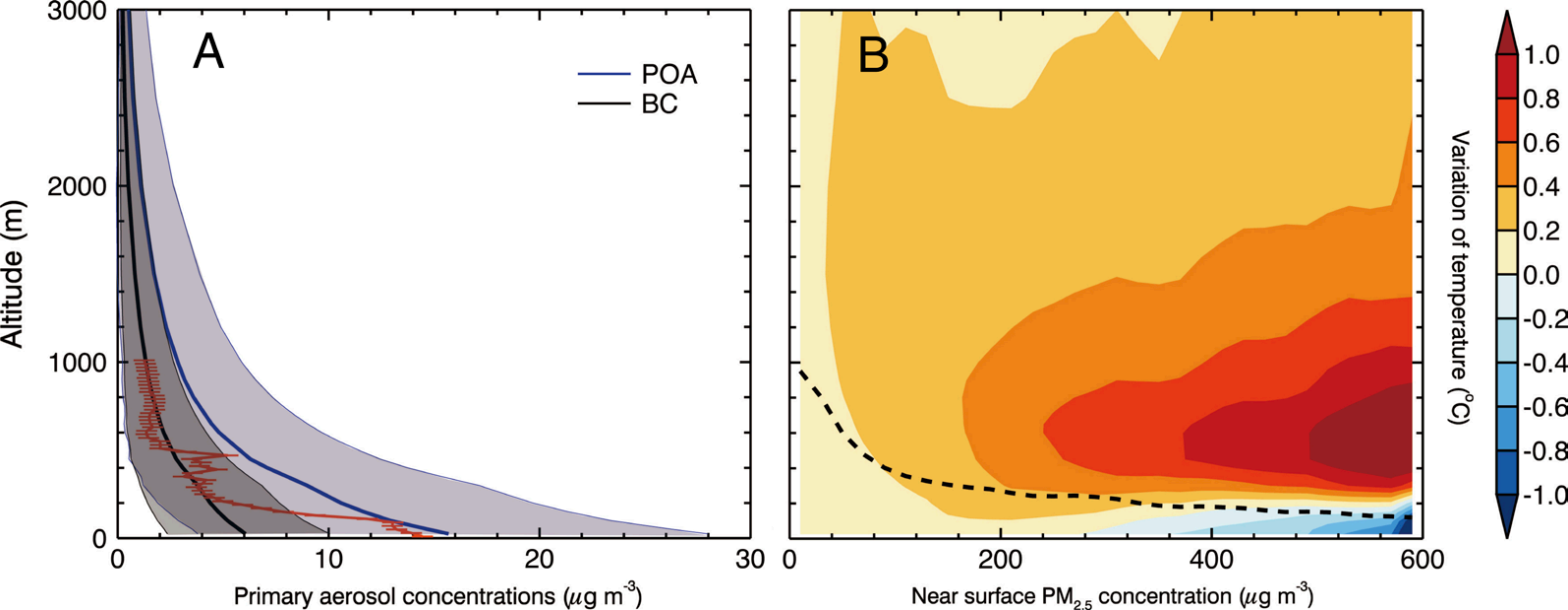
(*A*) Profile of average BC and POA concentrations with error shadow in F_BASE_ and (*B*) vertical distribution of the temperature variation due to AARI as a function of near-surface [PM_2.5_] in the NCP from 05 December 2015 to 06 March 2016. The dashed line in *B* represents the PBLH in F_BASE._ The red line with error bars indicates the vertical profile of observed daytime BC concentrations in Shijiazhuang from 13 to 16 January 2019 with the occurrence of a heavy haze event.

Vertical distributions of light-absorbing aerosols are crucial in evaluating the AARI and AAPI effects on PM pollution. From 05 December 2015 to 06 March 2016, the dominant light-absorbing aerosols, BC and primary organic aerosols (POA), are mainly concentrated in the NCP where highly dense industrial, traffic, and residential emissions are located (*SI Appendix*, Fig. S22), indicating the predominant local sources to form the haze. Fig. 2*A* presents the vertical profile of the BC and POA concentrations averaged during the study episode in the NCP. BC and POA concentrations show decreases with the altitude, with the maximum near the ground surface. The simulated BC profile in the study matches well with that observed using a tethered balloon system in Shijiazhuang of the NCP in January 2019 (Fig. 2*A*). Furthermore, during the wintertime in Beijing, aircraft observed BC vertical profiles over Beijing can be divided into two distinct categories (20). When local emissions dominate the air pollution in Beijing, the vertical distribution of BC is marked by a decreasing trend with increasing altitude. Conversely, the alternate type of BC vertical profile exhibits peak concentrations around 900 hPa, predominantly due to regional transport from the polluted areas to the south-southwest. The present results agree with many previous studies on the bottom-heavy BC vertical profiles in the NCP during the wintertime PM pollution using aircraft or tethered balloon-based BC measurements (21–23).

Different from the BC vertical profiles, the temperature increase caused by AARI peaks above the PBL top. The maximum temperature increase occurs at 400~700 m, in the range between 0.5 to 1.3 °C (Fig. 2*B*) when [PM_2.5_] rising from 200 to 600 μg m^−3^. On average, the AARI induced temperature enhancement peaks at around 900 m in the NCP during the episode, attaining about 0.30 °C (*SI Appendix*, Fig. S23). Such a phenomenon has also been found in previous studies (7, 24, 25). The temperature increase or heating rate caused by AARI is most significant above the PBL top, resulting in a “warm bubble” effect above the PBL top and facilitating upward motions to generate a secondary circulation. Although the vertical profile of absorbing aerosols (such as BC) reaches its maximum near the surface (Fig. 2), the heating rate peaks around 900 m, indicating that aerosol light absorption above the PBLH is more efficient. The heating rate experienced by a layer of air due to the absorption by aerosols for the i band can be expressed in height coordinates as (26):

[1] $${\left(\frac{\partial T}{\partial t}\right)}_{i}=-\frac{1}{\rho c_{p}}\frac{\partial F}{\partial z}=-\frac{1}{\rho (z)c_{p}}\frac{\Delta F}{\Delta z},$$

where ρ and $c_{p}$ denote the air density and specific heat at constant pressure, respectively. F is the net solar flux, which can be expressed as:

[2] $$F(z)=F^{↑}(z)+F^{↓}(z),$$

where $F^{↑}\left(z\right)$ and $F^{↓}\left(z\right)$ are the upward and downward solar flux for any given z within each layer. According to the Lambert-Beer law, the absorbed solar flux by light-absorbing aerosols at a given layer can be expressed as:

[3] $$\left|\Delta F\right|=\left(\left|F^{↓}\left(z+\Delta z\right)\right|+\left|F^{↑}(z)\right|\right)\left(1-e^{-\Delta \tau_{a}}\right),$$

where $\Delta \tau_{a}$ is the absorbing AOD (AAOD) for the given layer. Eq. **1** can be further expressed as:

[4] $$(\frac{\partial T}{\partial t}{)}_{i}=\frac{1}{\rho (z)c_{p}}\frac{\left(\left|F^{↓}\left(z+\Delta z\right)\right|+\left|F^{↑}(z)\right|\right)(1-e^{-\Delta \tau_{a}})}{\Delta z}.$$

When $\Delta \tau_{a}$ is small enough, Eq. **4** can be approximated as:

[5] $$(\frac{\partial T}{\partial t}{)}_{i}=\frac{1}{\rho (z)c_{p}}\left(\left|F^{↓}\left(z+\Delta z\right)\right|+\left|F^{↑}(z)\right|\right)\frac{\Delta \tau_{a}}{\Delta z}.$$

The heating rate induced by light-absorbing aerosols at a given layer with the fixed height (Δz) is determined by the upward and downward solar flux transferring the layer, the AAOD, and the air density of the layer. When the incident solar radiation transfers to the PBL, the air at the top of the PBL receives the maximal downward solar flux when comparing to below considering the aerosol scattering and absorbing in the PBL. At the same time, the aerosol scattering mainly occurs at the bottom of the PBL based on the aerosol vertical profile, so the upward solar flux at the top of the PBL is also maximum in the PBL (*SI Appendix*, Fig. S24*A*). With the sufficient atmospheric aging process and the atmospheric diffusion, the absorbing aerosols are subject to being mixed internally with other aerosols. The reduction in AAOD at the top of PBL is merely 40% relative to its value close to the surface layer (*SI Appendix*, Fig. S24*B*), which is much slower than the decrease of light-absorbing aerosol concentrations (Fig. 2). The air density also decreases by 5% at the top of PBL compared to its value near the surface (*SI Appendix*, Fig. S24*C*). Furthermore, AARI also leads to significant reduction in solar radiation reaching the ground, resulting in the most noticeable cooling at the surface. The higher the height, the less the air is affected by the surface cooling, leading to more pronounced warming compared to the lower atmosphere. Hence, if the AARI was not considered, the maximum temperature increase caused by absorbing aerosols should appear at the top of the PBL where vertical motions could carry the particulates. However, the development of PBL is inhibited by the feedback of AARI through enhancing the atmospheric stability (*SI Appendix*, Fig. S24*A*). Therefore, the maximum heating rate is pronounced above the PBL due to the decreased PBLH. AARI generally causes a slight warming of the near-surface atmosphere when [PM_2.5_] is below 280 μg m^−3^, whereas it leads to a decrease in near-surface temperature when [PM_2.5_] exceeds 280 μg m^−3^ (Fig. 2*B*). Temperature decrease can be as large as 0.5 °C when PM_2.5_ concentrations exceed 400 μg m^−3^. Therefore, the temperature inversion caused by AARI increases with increasing [PM_2.5_] in the PBL. Atmospheric warming in the upper level coupled with surface cooling significantly alters the daily vertical temperature gradient, consequently affecting atmospheric stability. As shown in *SI Appendix*, Fig. S25, the warming at the upper level in the afternoon and cooling effect near the surface in the morning due to AARI are clearly identified. Since midday (14:00 Local Time), the maximal upper-level heating exceeds 0.3 °C around 900 m owing to the intense shortwave radiation and the accumulated heating effect before midday. The cooling effect near the surface appears in the morning (08:00 to 11:00 Local Time) and decreases rapidly with the PBL development in the late morning. In the evening, with the enhanced atmospheric stability, the PBLH presents a decreasing trend. AARI induced thermal stratification across diverse atmospheric levels markedly perturbs atmospheric stability, consequently influencing the vertical profile of PM_2.5_.

**Fig. 3.**
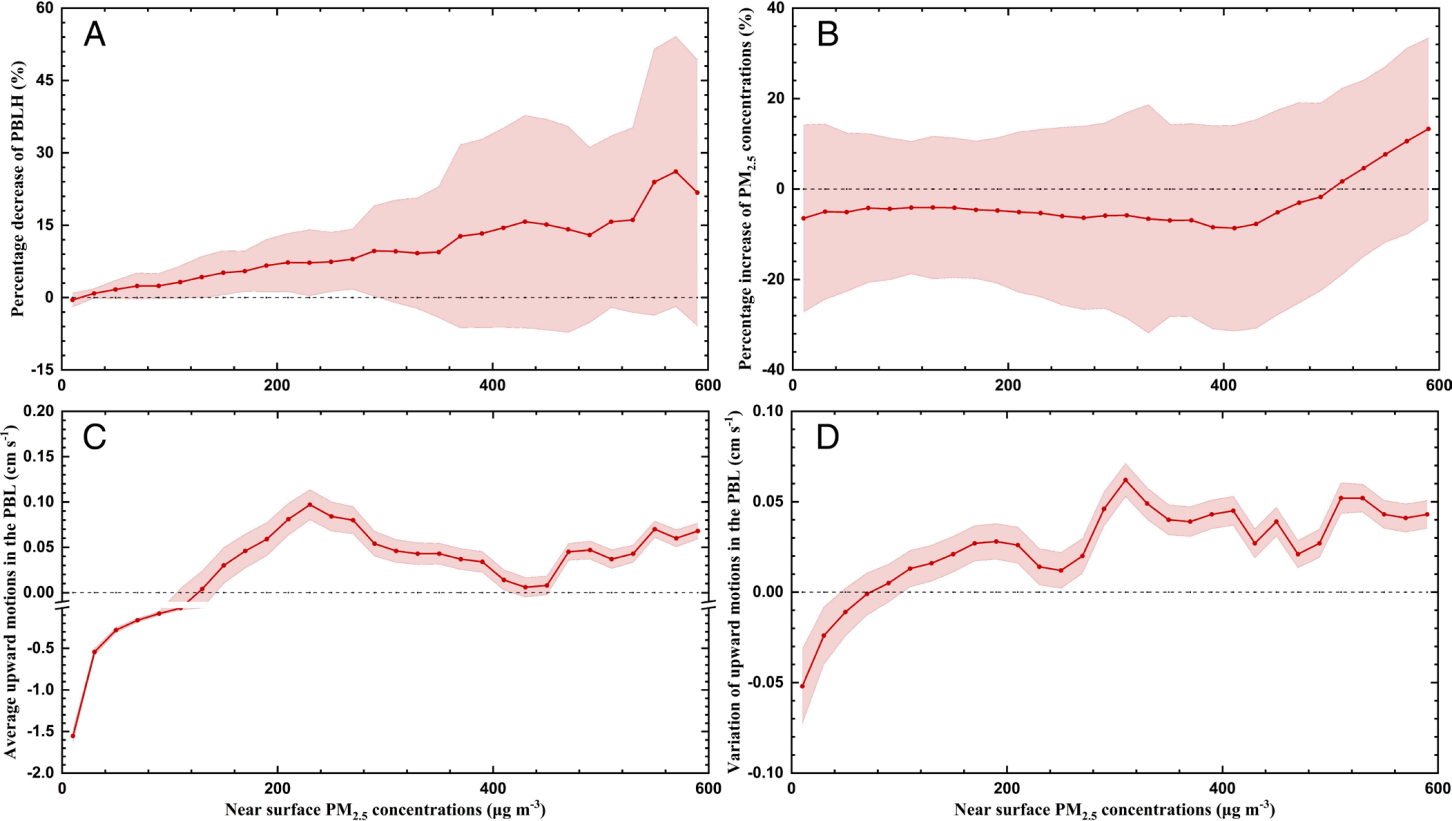
(*A*) Decrease in PBLH and (*B*) increase in PM_2.5_ concentrations caused by AARI, (*C*) average upward motions in the PBL in F_AARI0_, and (*D*) AARI induced changes in upward motions in the PBL with error shadow (range of SD) as a function of the near-surface [PM_2.5_] in the NCP from 05 December 2015 to 06 March 2016.

The uneven heating rate caused by light-absorbing aerosols at different levels enhances the temperature inversion near the surface and suppresses the PBL development. Fig. 3*A* shows the decrease in PBLH attributable to AARI with increase of [PM_2.5_]. AARI tends to decrease the PBLH in all conditions. With [PM_2.5_] below 400 μg m^−3^, the PBLH generally exhibits a linear decline with rising PM_2.5_ levels. The PBLH is decreased more substantially by 15 to 30% with [PM_2.5_] rising from 500 to 600 μg m^−3^ due to the pronounced temperature inversion caused by AARI (Fig. 2*B*). The present simulations also show that AARI inhibits PBL development with a decrease of PBLH by 2.4% or 11.7 m (*SI Appendix*, Fig. S26), such a magnitude is smaller than previous reports (7, 25, 27, 28), likely because we investigate a larger domain on the regional scale, rather than the city scale. When considering the AARI effect at a large spatiotemporal scale, as the whole NCP in this study, near-surface [PM_2.5_] are not increased at a constant rate as expected by following the corresponding decrease of the PBLH. Only when [PM_2.5_] rise from 500 to 600 μg m^−3^, AARI enhances [PM_2.5_] by about 2 ~ 15% (Fig. 3*B*). When the [PM_2.5_] are below 500 μg m^−3^, AARI reduces [PM_2.5_] by about 2 ~ 9%. On average, AARI alleviates the PM pollution near the surface, reducing [PM_2.5_] by 4.9 μg m^−3^ or 4.6% (*SI Appendix*, Fig. S27). After the haze forms, the PM_2.5_ profile is determined mainly by vertical dispersion and transport. Therefore, the decrease in near-surface [PM_2.5_] by AARI could be attributable to the adjustment of vertical motions at a large scale.

The vertical motion over the PM pollution region in North China typically exhibits a pattern of ascending–descending–ascending distribution, with the wind directions showing a sequence of convergence–divergence–convergence from surface up to the mid-levels of the troposphere (29, 30). The shallow convergence below 900 hPa is conducive to the accumulation of air pollution near the surface. A vertical sinking motion at 900 to 700 hPa is a critical dynamic mechanism for the formation of a persistent haze event by affecting the PBLH to inhibit the dilution of air pollutants (*SI Appendix*, Fig. S28). The vertical profile of wind divergence during the study episode is analyzed during the heavy haze episodes (*SI Appendix*, Fig. S29). The wind divergence shows the above-mentioned structure of a convergence below 900 hPa, a divergence in the middle-lower troposphere (900 to 700 hPa), and a convergence in the middle-upper troposphere over the PM pollution area. *SI Appendix*, Fig. S30 shows the proportion of PM pollution days (hourly PM_2.5_ concentration > 75 μg m^−3^ and lasing two or more days) with average PM_2.5_ concentrations during the wintertime from 2013 to 2023. After the implementation of the “Atmospheric Pollution Prevention and Control Action Plan” since September 2013, the occurrence of PM pollution in the NCP presents a decreasing trend, and the intensity of PM pollution also shows a downward trend, with the average PM_2.5_ concentrations ranging from 115 to 250 μg/m^3^. In the previous study by Wu et al. (29), the occurrence frequency of regional persistent haze events per year from 1980 to 2013 has been identified with an increasing tendency with the above-mentioned dynamic structure.

Fig. 3*C* provides the average upward /downward motions in the PBL (below 900 hPa) as a function of [PM_2.5_] bins in the NCP in the F_AARI0_. The downward airflow dominates the area with near-surface [PM_2.5_] below 120 μg m^−3^, and it is opposite for the area with near-surface [PM_2.5_] exceeding 120 μg m^−3^. Therefore, the relatively clean or divergence area is commonly influenced by sinking airflow, and the polluted or convergence area corresponds to the weak upward airflow. As shown in Fig. 3*D*, the heating effect caused by AARI induces a descending motion in the divergence area and an ascending motion in the convergence area. Hence, in the convergence area, the upward motion is enhanced by AARI, facilitating the lifting of air pollutants from the ground surface to the higher level to lower [PM_2.5_] near the surface. In the divergence area, the enhanced downward motion drags cleaner air from the higher level, decreasing near-surface [PM_2.5_]. *SI Appendix*, Fig. S31 shows the spatial variations of distribution of upward motions in the PBL and changes of temperature at around 900 m during the polluted episode with occurrence of heavy PM pollution ([PM_2.5_] > 250 μg m^−3^) selected from 05 December 2015 to 06 March 2016. When the heavy PM pollution occurs, enhanced upward motions appear in Eastern China with [PM_2.5_] near the surface above 75 μg m^−3^. The spatial distribution of temperature increases at around 900 is well consistent with the spatial characteristics of the enhanced upward motions in the PBL caused by AARI. At a large scale, AARI induces a secondary circulation in Eastern China, facilitating the convergence in the NCP (*SI Appendix*, Fig. S32*A*). AARI increases the convergence with [PM_2.5_] above 75 μg m^−3^ and amplifies the divergence when [PM_2.5_] is less than 75 μg m^−3^ (*SI Appendix*, Fig. S32*B*), which is in accordance with the changes in the descending/ascending motions. The variation of near-surface [PM_2.5_] reflects a synergetic effect of the vertical dispersion and upward motion caused by AARI. When PM pollution becomes severe with [PM_2.5_] above 500 μg m^−3^, the effect of the PBLH decrease due to temperature inversion overweighs the warm bubble effect, leading to an elevation of the PM pollution.

Here the reported turning point at [PM_2.5_] of 500 μg m^−3^ qualitatively agrees with an idealized simulation study (31) that also identified a turning point at AOD of 1.3, above which the maximum PBLH decreases rapidly to aggravate the air pollution with the strengthened vertical stability. They also pointed out that the strong mixing effect induced by the light absorption below the “turning point” AOD can decrease the air pollutants at the surface by upward transport. However, our results show that the reduction in PM_2.5_ is primarily attributed to the intensified upward motion induced by AARI with [PM_2.5_] below 500 μg m^−3^. Climate model simulations have also revealed that absorbing aerosols over Asia intensify low-altitude convergence and vertical airflow, further overcoming the stabilizing effects of absorbing aerosols and enhancing the summer monsoonal circulation (9). Li et al. (32, 33) have also reported that the interaction between absorbing aerosols and radiation has the potential to modify the thermodynamic stability and convective activity in the lower atmosphere, thereby intensifying the onset of the early monsoon and influencing its subsequent evolution. Absorbing aerosols may contribute to atmospheric instability, leading to regional warming and increased flooding incidents, and are crucial in the escalation of pollution events (3, 9, 34, 35). Therefore, the above findings also support the result in this study that AARI can increase the vertical movement on a large scale to reduce near-surface [PM_2.5_].

The aerosol light absorption may change the cloud structure and thermal contrast between land and sea to disturb atmospheric circulation on a global scale. During the winter season, North China is affected by East Asian winter monsoon with northwesterly winds. A global modeling study by Lou et al. (36) has found that the midlatitude BC emissions create an anomalous thermal contrast between land and sea, resulting in a reduction of the zonal component of the prevailing northwesterly winds of the East Asian winter monsoon, thus aggravating the PM pollution by weakening the East Asian winter monsoon. The study attributes the aggravation of PM pollution in North China to the decreased wind speed due to the changed atmospheric circulation induced by BC. The wind speeds decrease by 0.024 (±0.007) m s^−1^ in Beijing per unit (1 Tg C) of midlatitude BC emissions, and the conclusion from Lou et al. (36) has also mentioned that the increase in BC emissions in China could be one possible contributor to decreased wind speed. The previous study by Xue et al. (37) reveals that the BC forcing leads to a significant warming of the upper-level atmosphere, thereby elevating temperatures over Asia region, which influences the dynamics of the upper-level westerlies and the circulation of the Asian summer monsoon, affecting associated precipitation patterns under scenarios with 10 times modern global BC emissions or concentrations. However, the tenfold increase in BC will reduce the Northern Hemisphere mid-latitudes precipitation mainly in summer. The feedbacks of the wintertime circulation and its variability on a global scale are not still considered in this study and further studies are needed in the future. However, it should be noted that the GCM generally exhibits limited precision in simulating regional dynamics and thermal conditions due to its coarse spatial resolution, which consequently impedes the accurate determination of heating rates caused by AARI.

It should be noted that the AARI effect not only alters the aerosol concentration and distribution in the PBL, also perturbs the temperature and wind field, which could influence clouds and radiation. However, the AARI effect increases slightly the low-level cloud optical thickness (COT) and cloud fraction (CF), with an average increase of 2.4% and 1.4% (*SI Appendix*, Fig. S33) over the NCP throughout the episode. It should be highlighted that the increase of COT and CF is more obvious during the nighttime than daytime, with the enhancement of 3.1% and 2.1%, respectively. The increased low-level clouds could intensify the AARI effect during the nighttime through the heating effect of cloud absorption of the longwave radiation from the surface.

**Fig. 4.**
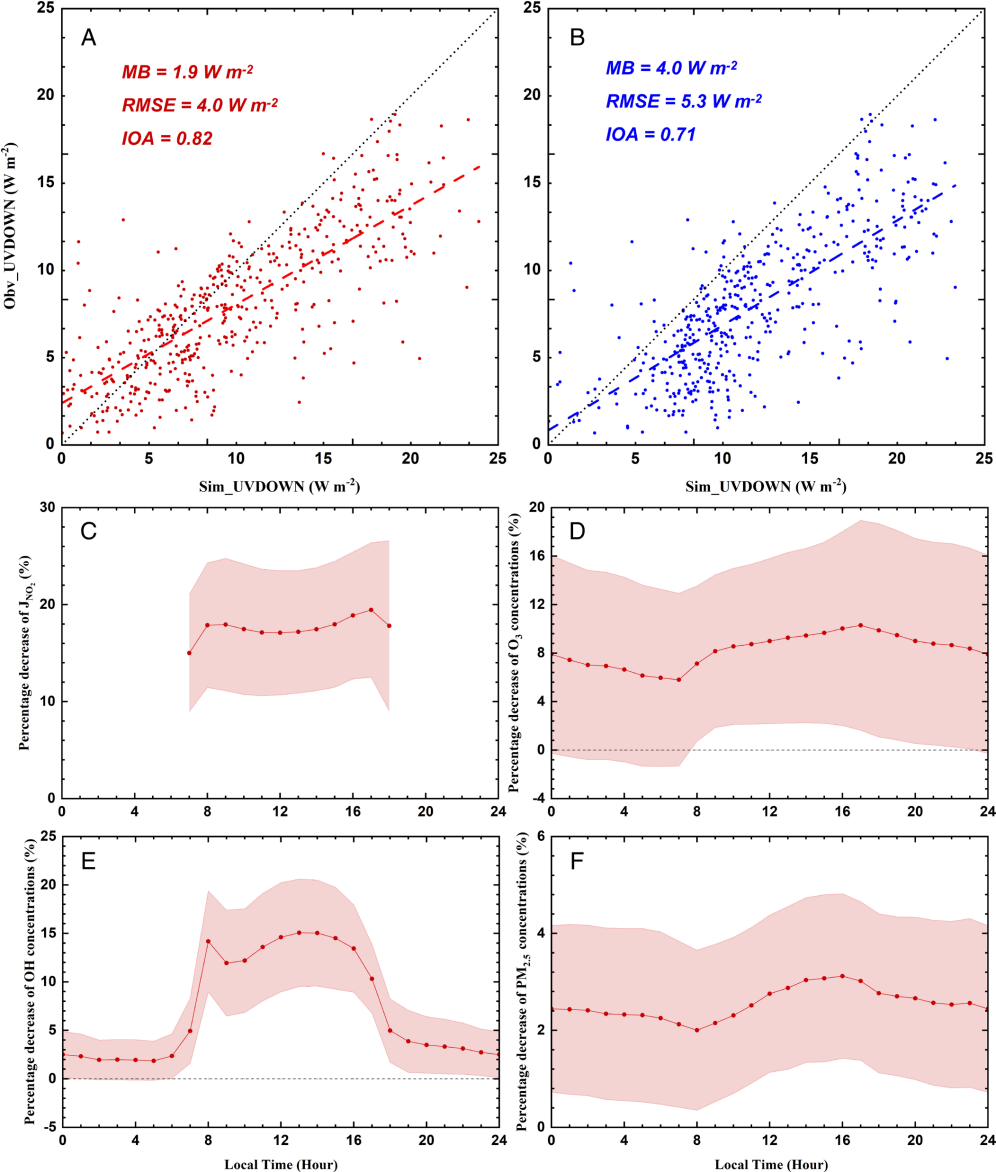
Scatter plots of observed and simulated daily profiles of the UVDOWN in *A* F_BASE_ (red dots) and (*B*) F_Abs0_ (blue dots) reaching the ground surface, and percentage decrease of diurnal variations of (*C*) $J_{{\mathrm{NO}}_{2}}$, (*D*) O_3_ concentrations, (*E*) OH concentrations, and (*F*) PM_2.5_ concentrations caused by AAPI with error shadow (range of SD) in the NCP from 05 December 2015 to 06 March 2016.

In addition to effects of AARI, absorbing aerosols-induced weakening of UV radiation certainly diminishes photolysis rates, thereby lowering ozone concentrations and impeding the formation of secondary aerosols (14, 15). The simulated temporal variation of UV radiation (wavelength at 200 ~ 420 nm) reaching the surface (UVDOWN) is well consistent with measurements at the five observation sites in the NCP (*SI Appendix*, Fig. S15). The simulated UVDOWN in F_BASE_ generally correlates well with that observed, with the IOA and MB of 0.82 and 1.9 W m^−2^, respectively (Fig. 4*A*). The predicted daily UVDOWN in F_Abs0_ exits obvious overestimations compared with observations, with the MB of 4.0 W m^−2^ (Fig. 4*B*). Comparing F_BASE_ with F_Abs0_, atmospheric absorbing aerosols significantly diminish the UV radiation, leading to a 20.3% decrease in UVDOWN during the episode in the NCP. In the troposphere, ground state oxygen atoms (O^3^P), generated from the photolysis of NO_2_, react with oxygen molecules (O_2_), serving as a crucial source of ozone (O_3_). Therefore, the NO_2_ photolysis rate is crucial in changing O_3_ concentration. Fig. 4 *C* and *D* show the diurnal cycle of average near-surface NO_2_ photolysis rate ($J_{{\mathrm{NO}}_{2}}$), O_3_, OH, and PM_2.5_ concentrations due to AAPI in the NCP during the episode. AAPI decreases $J_{{\mathrm{NO}}_{2}}$ effectively by 14 ~ 20% during daytime (Fig. 4*C*), and correspondingly O_3_ concentration ([O_3_]) is reduced by 6 ~ 10% (Fig. 4*D*). In addition, $J_{{\mathrm{NO}}_{2}}$ and [O_3_] generally decrease linearly with increasing [PM_2.5_]. With [PM_2.5_] increasing from 100 to 600 μg m^−3^, the decrease of $J_{{\mathrm{NO}}_{2}}$ and [O_3_] ranges from 6.5 to 52% and from 0.3 to 61%, respectively (*SI Appendix*, Fig. S34). AAPI decreases the average near-surface $J_{{\mathrm{NO}}_{2}}$ and [O_3_] by about 16.5% and 8.3% in the NCP (*SI Appendix*, Fig. S35). Consequently, near-surface daytime OH concentrations ([OH]) are decreased by more than 10% (Fig. 4*E*), particularly with [PM_2.5_] above 200 μg m^−3^, the decrease exceeds 20%. AAPI reduces the average near-surface [OH] by 14.2% in the NCP during the daytime.

Pronounced reductions of O_3_ and OH, the primary oxidants in the atmosphere, due to AAPI inevitably decrease secondary aerosol formation. *SI Appendix*, Fig. S36 presents the spatial variations of secondary aerosols owing to AAPI during the episode. Since heterogeneous reactions involving aerosol water play a dominant role in wintertime sulfate level (38, 39), AAPI does not appreciably alter the sulfate formation in the NCP, reducing sulfate aerosols by 0.5 μg m^−3^ or 3.7% on average. As the nitrate precursors, formation of HNO_3_ and N_2_O_5_ is mainly determined by OH and O_3_, respectively, so the decrease of nitrate concentrations due to AAPI is noticeable, with an average of 0.9 μg m^−3^ or 4.1%. The ammonium concentration in the NCP is also decreased by 0.3 μg m^−3^ or 3.3% on average due to the decrease in sulfate and nitrate. Although the wintertime AOC is low, the SOA formation is still sensitive to the AOC (40, 41). The reduction in SOA concentrations attributed to AAPI is substantial, reaching 1.4 μg m^−3^ or 9.2%. In total, AAPI decreases near-surface [PM_2.5_] by 3.4 μg m^−3^ or 3.1% (*SI Appendix*, Fig. S37), and the PM_2.5_ decrease fluctuates around 3% (Fig. 4*F*).

The present modeling results further show a linear additivity of two effects, AARI and AAPI. On average, AARI decreases near-surface [PM_2.5_] by 4.9 μg m^−3^, and the PM_2.5_ decrease due to AAPI is 3.4 μg m^−3^. When both effects are simultaneously excluded in a sensitivity experiment, the absence of AARI and AAPI increases [PM_2.5_] by 8.1 μg m^−3^ (*SI Appendix*, Figs. S38 and S39), which is approximately equal to the summation (8.3 μg m^−3^) of that caused by only AARI and AAPI. Such a net effect makes the light-absorbing aerosols more relevant than the purely scattering aerosols in providing feedbacks to surface PM formation and accumulation.

## Concluding Remark

**Fig. 5.**
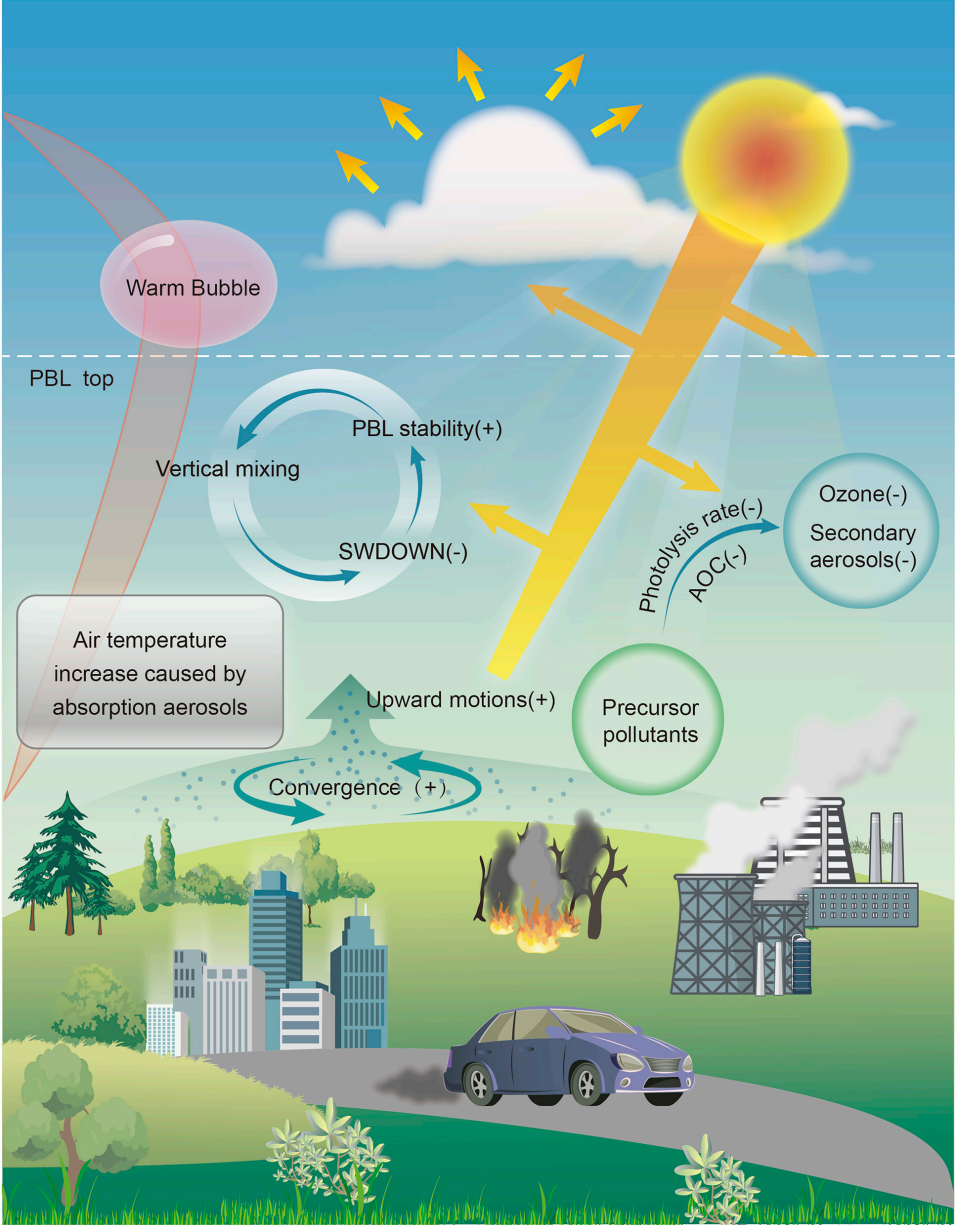
Effects of absorption of aerosols on PM pollution development by interactions with solar radiation and photolysis in winter. The dome effect caused by the aerosol absorption inhibits the PBL development to cause the PM pollution deterioration at the surface level. The warm bubble effect caused by the most significant heating rate above the PBL top enhances the upward motions in the convergence or polluted area and the downward motions in the divergence or relatively clean area. The upward motion is enhanced in the convergence area by AARI, facilitating the lifting of air pollutants from the ground surface to the higher level to lower near-surface [PM_2.5_]. The enhanced downward motion in the divergence brings more clean air from the higher level to decrease near-surface [PM_2.5_]. In addition, aerosol absorption effectively decreases photolysis to lower the AOC and inhibit ozone formation, reducing the secondary aerosol concentration.

Here we identify that the positive contribution of aerosol light absorption to particulate accumulation and haze formation is likely overestimated in the previous literature. The mechanisms identified are summarized in the schematics in Fig. 5. Absorption of aerosols like BC can generate an uneven heating rate in the vertical direction. During the severe haze with stagnant air and low PBLH, the heating rate is the largest right above the PBL top, which causes a temperature inversion in the PBL and hinders the PBL development and vertical dispersion of air pollutants. The present model simulations successfully reproduce the vertically uneven heating rate caused by absorbing aerosols and a corresponding decrease in PBLH. We further find that near-surface [PM_2.5_] is decreased, leading to the mitigation of PM pollution. The warm bubble effect caused by the pronounced aerosol absorption above the PBL generates a secondary circulation, enhancing the upward motion and horizontal convergence in the polluted area and the downward motion and divergence in the relatively clean area. Additionally, aerosol absorption effectively suppresses NO_2_ photolysis to hinder the O_3_ formation and lower the AOC, inhibiting the secondary aerosol formation. The total effect of aerosol absorption, or the synergetic effect of AARI and AAPI, decreases near-surface [PM_2.5_] by 8.1 μg m^−3^ or 7.4%.

Our results suggest that distinctive effects of aerosol absorption should be comprehensively accounted for when developing the mitigation strategies to reduce the severity of wintertime haze events. BC is the major contributor to aerosol absorption and accounts for about 5.6 μg m^−3^ of near-surface [PM_2.5_] as a PM_2.5_ constituent, even lower than the PM_2.5_ decrease caused by BC-related light absorption. Therefore, the presence of BC can result in a net reduction of the surface PM concentration. The ascending motion of the warm bubble effect near the PBL top due to aerosol absorption facilitates more intensive vertical development of cloud formation, which may partially cancel out the warming effect. Furthermore, aerosol absorption decreases photolysis to lower the AOC, hindering the formation of secondary aerosols and slowing the aerosol nucleation process. Such a mechanism has further potentials of reducing cloud condensation nuclei for aerosol–cloud interactions. Therefore, light-absorbing aerosols increase the complexity of physical and chemical processes in the numerical models and need sufficient treatment in pollution, weather, and climate predictions.

## Materials and Methods

Quality-assured ground-based datasets of columnar aerosol optical properties, including AOD and SSA at 440, 675, 870, and 1,020 nm were retrieved from the long-term measurements of a Cimel 318-NE Sun-sky radiometer at five typical observation sites in the NCP, with locations of urban Beijing, Institute of Remote Sensing and Digital Earth, Chinese Academy of Sciences (Institute of Remote Sensing and Digital Earth (RADI)) in Beijing, Chinese Academy of Meteorological Sciences observation site in Beijing, Xianghe, and Xinglong (*SI Appendix*, Fig. S1*B*). The measurements of hourly PM_2.5_ concentrations adopted in the present study were provided by China’s Ministry of Environment Protection. The WRF-Chem model (Version 3.5) used in this study is updated by Li et al. (15, 42, 43) to quantitatively assess the impacts of AARI and AAPI on [PM_2.5_] with occurrence of a severe haze event. The detailed model descriptions and configuration can be found in *SI Appendix*.

## Supplementary Material

Appendix 01 (PDF)

## Data Availability

All study data are included in the article and/or *SI Appendix*. All the shared data have been deposited in a publicly available database, including a direct link to the data (44).
