# Supplementary material for: Aerosol light absorption alleviates particulate pollution during wintertime haze events
Source: Proc Natl Acad Sci U S A. 2024 Dec 23;122(1):e2402281121. doi: 10.1073/pnas.2402281121 (PMC11725903; doi:10.1073/pnas.2402281121)
Supplement: Supplementary file 1 — Appendix 01 (PDF) [file pnas.2402281121.sapp.pdf]

PNAS

Supporting Information for

Aerosol light absorption alleviates particulate pollution during wintertime haze events

Jiarui Wu *et al.*

Corresponding author(s): Guohui Li, ligh@ieecas.cn; Yuan Wang, yzwang@stanford.edu; John H. Seinfeld, seinfeld@caltech.edu

This PDF file includes:

Materials and Methods
Model Validation
Tables S1 and S2
Figs. S1 to S39
SI References

Materials and Methods

WRF-Chem model. The WRF-Chem model (Version 3.5) (*1*) with modifications by Li et al. (*2-4*) has been applied to quantitatively evaluate the $PM_{2.5}$ contribution of the combination of ARI and API during a persistent heavy haze episode in the NCP. The model includes a new flexible gas phase chemical module and the CMAQ aerosol module developed by US EPA (*5*). For the aerosol simulations, the CMAQ/models-3 aerosol module (AERO5) has been incorporated into the model. In this aerosol component, the particle size distribution is represented as the superposition of three lognormal sub-distributions, called modes. The processes of coagulation, particles growth by the addition of mass, and new particle formation are included. The new particle production rate due to binary nucleation of H_2SO_4 and water vapor is parameterized following Kulmala et al. (*6*). The wet deposition is based on the method in the CMAQ module and the dry deposition of chemical species followed Wesely (*7*). The photolysis rates are calculated using the Fast Tropospheric Ultraviolet and Visible (FTUV) Radiation Model with the aerosol and cloud effects on photolysis (*3, 8*).

ISORROPIA (version 1.7) is used to predict the thermodynamic equilibrium between the ammonia-sulfate-nitrate-chloride-water aerosols and their gas phase precursors of H_2SO_4-HNO_3-NH_3-HCl-water vapor (*9*). The organic aerosol (OA) module is based on the volatility basis-set (VBS) approach with aging; detailed information can be found in Li et al. (*4*). The primary OA (POA) components from traffic-related combustion and biomass burning emissions are represented by nine surrogate species with saturation concentrations (C*) ranging from 10^{-2} to 10^{6} μg m^{-3} at room temperature (*10*), and assumed to be semi-volatile and photochemically reactive (*11*). The secondary OA (SOA) formation from each anthropogenic or biogenic precursor is calculated using four semi-volatile VOCs with effective saturation concentrations of 1, 10, 100, and 1000 μg m^{-3} at 298 K. The SOA formation via the heterogeneous reaction of glyoxal and methylglyoxal is parameterized as a first-order

irreversible uptake by aerosol particles and cloud droplets with an uptake coefficient of 3.7×10^{-3} (*12-14*).

Model configurations. In the Base scenario simulation, the physical parameterizations include the microphysics scheme of Hong et al. (*15*), the Mellor, Yamada, and Janjić (MYJ) turbulent kinetic energy (TKE) planetary boundary layer scheme (*16*), the Unified Noah land-surface model (*17*), the Goddard longwave radiation scheme (*18*) and the Goddard shortwave parameterization (*19*). The NCEP 1°× 1° reanalysis data are used to obtain the meteorological initial and boundary conditions. The chemical initial and boundary conditions are interpolated from the 6h output of MOZART (*20*). The spin-up time of the WRF-Chem model is 4 days and 4 hours. The SAPRC-99 (Statewide Air Pollution Research Center, version 1999) chemical mechanism is used in the present study. The anthropogenic emissions are developed by Zhang et al. (*21*) and Li et al. (*22*), including contributions from agriculture, industry, power generation, residential, and transportation sources. The biogenic emissions are calculated online using the MEGAN (Model of Emissions of Gases and Aerosol from Nature) model developed by Guenther et al. (*23*). The Fire Inventory from NCAR (FINN) (*24, 25*) is taken for the biomass burning emissions in simulations. The model simulation domain is shown in Fig. S1a and detailed model configuration can be found in Table S1.

Aerosol radiative module. In the present study, Goddard shortwave module developed by Chou and Suarez (*18, 19*) is employed to account for the ARI effect on particulate matter (PM) pollution and the FTUV module (*3, 8*) is used to consider the API effect. The aerosol radiative module developed by Li et al. (*3*) has been incorporated into the WRF-Chem model to calculate the aerosol optical depth (AOD or $\boldsymbol{\tau_a}$), single scattering albedo (SSA or $\boldsymbol{\omega_a}$), and the asymmetry factor ($\boldsymbol{g_a}$).

In the CMAQ aerosol module, aerosols are represented by a three-moment approach with a lognormal size distribution:

$$\boldsymbol{n(lnD)} = \frac{\boldsymbol{N}}{\sqrt{\boldsymbol{2\pi}}\boldsymbol{ln\sigma_g}}\boldsymbol{exp}[-\frac{1}{2}(\frac{\boldsymbol{lnD-lnD_g}}{\boldsymbol{ln\sigma_g}})^2] \quad (1)$$

Where D is the particle diameter, N is the number distribution of all particles in the distribution, D_g is the geometric mean diameter, and σ_g is the geometric standard deviation. To calculate the aerosol optical properties, the aerosol spectrum is first divided into 48 bins from 0.002 to 20.0 µm, with radius r_i. The aerosols are classified into four types: (1) internally mixed sulfate, nitrate, ammonium, hydrophilic organics and black carbon (BC), and water; (2) hydrophobic organics; (3) hydrophobic BC; and (4) other unidentified aerosols (generally dust-like aerosols). These four kinds of aerosols are assumed to be mixed externally. For the internally mixed aerosols, the complex refractive index at a certain wavelength (λ) is calculated based on the volume-weighted average of the individual refractive index. Given the particle size and complex refractive index, the extinction efficiency (Q_e), ω_a and g_a are calculated using the Mie theory at a certain wavelength (λ). The look-up tables of Q_e, ω_a and g_a are established according to particle sizes and refractive indices to avoid multiple Mie scattering calculation. The aerosol optical parameters are interpolated linearly from the look-up tables with the calculated refractive index and particle size in the module.

The $\boldsymbol{\tau_a}$ at a certain $\boldsymbol{\lambda}$ in a given atmospheric layer $\boldsymbol{k}$ is determined by the summation over all types of aerosols and all bins:

$$\boldsymbol{\tau_a(\lambda,k) = \sum_{i=1}^{48}\sum_{j=1}^{4} Q_e\,(\lambda, r_i, j, k)\pi {r_i}^2 n(r_i, j, k)\Delta Z_k} \quad (2)$$

where $\boldsymbol{n(r_i, j, k)}$ is the number concentration of $\boldsymbol{j}$-th kind of aerosols in the $\boldsymbol{i}$-th bin. $\boldsymbol{\Delta Z_k}$ is the depth of an atmospheric layer. The weighted-mean values of $\boldsymbol{\omega_a}$ and $\boldsymbol{g_a}$ are then calculated by using d'Almeida et al. (*26*):

$$\boldsymbol{\omega_a(\lambda,k) = \frac{\sum_{i=1}^{48}\sum_{j=1}^{4} Q_e(\lambda, r_i, j, k)\pi {r_i}^2 n(r_i, j, k)\omega_a(r_i, j, k)\Delta Z_k}{\sum_{i=1}^{48}\sum_{j=1}^{4} Q_e(\lambda, r_i, j, k)\pi {r_i}^2 n(r_i, j, k)\Delta Z_k}} \quad (3)$$

$$g_a(\lambda,k) = \frac{\sum_{i=1}^{48}\sum_{j=1}^{4} Q_e(\lambda,r_i,j,k)\pi r_i^2 n(r_i,j,k)\omega_a(r_i,j,k) g_a(\lambda,r_i,j,k)\Delta Z_k}{\sum_{i=1}^{48}\sum_{j=1}^{4} Q_e(\lambda,r_i,j,k)\pi r_i^2 n(r_i,j,k)\omega_a(r_i,j,k)\Delta Z_k} \quad (4)$$

When the wavelength-dependent τ_a, ω_a, and g_a are calculated, they can be used in the Goddard shortwave module to evaluate the ARI effect and the FTUV to evaluate the API effect. The aerosol refractive indices used for Mie scattering calculation are listed in Table S2. In the base case simulation of the Base scenario, the BC aging from the hydrophobic to the hydrophilic state occurs at a pseudo first order rate of 9.26×10^{-5} s^{-1} (*27*) during daytime and 7.10×10^{-6} s^{-1} (*28*) during nighttime. As suggested by Moffet and Prather (*27*), the effective density is 0.7 g cm^{-3} for fresh BC and 1.8 g cm^{-3} for aged BC to consider the variation of the BC morphology. In order to take into account absorption of brown carbon (BrC) observed by Barnard et al. (*29*) in Megacities, the imaginary refractive index of POA measured by Kirchstetter et al. (*30*) is employed in the present study (Table S2). Detailed information can be found in Li et al. (*3*).

Data description. The model performance is validated using the available measurements in the NCP, including AOD, SSA, planetary boundary layer height (PBLH), downward shortwave flux (SWDOWN), aerosol species, and air pollutants. The AOD data retrieved from OMI (Ozone Monitoring Instrument) are used for the model validation of the spatial distribution of AOD. The hourly SSA at 520nm used for model validation is calculated using the measurement of the turbidity meter at the National Center for Nanoscience and Technology (NCNST), Chinese Academy of Sciences (116.33°E, 39.99°N) in Beijing (Fig. S1b). The long term measurements of Quality-assured ground-based datasets of SSA at 440 nm at Institute of Remote Sensing and Digital Earth (RADI), Chinese Academy of Sciences in Beijing are also used for the validation of the three-month model simulation. The daily PBLH at 12:00 Beijing time (BJT) is diagnosed from the radiosonde observation at a meteorological site (116.47°E, 39.81°N) in Beijing. The hourly measurements of O_3, NO_2, SO_2, CO and $PM_{2.5}$ concentrations

have been released by the Ministry of Ecology and Environment of China since 2013. The hourly submicron sulfate, nitrate, ammonium, and organic aerosols are measured by the Aerodyne Aerosol Chemical Speciation Monitor (ACSM) at NCNST. The hourly black carbon (BC) concentration is also measured using a thermal/optical reflectance carbon analyzer (OCEC RT-4, Sunset Lab, USA) at NCNST site in Beijing. Comprehensive observation campaigns with vertical profile of BC concentrations using a tethered balloon system within the lower troposphere (1 km) are carried out at the Yuanshi National Meteorological Station (114.5°E, 37.8°N) in the south of Shijiazhuang in January 2019 (*31*). The SWDOWN is measured by CM-11 pyranometers at five sites from Chinese Ecosystem Research Network (CERN) in the NCP, including Beijing, Luancheng, Yucheng, Jiaozhouwan, and Fengqiu (Fig. S1b) (*32*). The observed meteorological parameters at seven meteorological sites (Fig. S1b) in the NCP are used for model validation, including 2m temperature (T2), relative humidity (RH), 10m wind speed (WS10), and wind direction (WD).

Statistical metrics for observation-model comparison. In the present study, the mean bias (*MB*), root mean square error (*RMSE*), the index of agreement (*IOA*), and correlation efficient (*R*) are used as indicators to evaluate the performance of WRF-Chem model in simulations against measurements. *IOA* describes the relative difference between the model and observation, ranging from 0 to 1, with 1 indicating perfect agreement.

$$\boldsymbol{MB} = \frac{1}{N}\sum_{i=1}^{N}(P_i - O_i) \quad (5)$$

$$\boldsymbol{RMSE} = \left[\frac{1}{N}\sum_{i=1}^{N}(P_i - O_i)^2\right]^{\frac{1}{2}} \quad (6)$$

$$\boldsymbol{IOA} = 1 - \frac{\sum_{i=1}^{N}(P_i - O_i)^2}{\sum_{i=1}^{N}(|P_i - \bar{O}| + |O_i - \bar{O}|)^2} \quad (7)$$

$$\boldsymbol{R} = \frac{\sum_{i=1}^{N}[(P_i - \bar{P}) \times (O_i - \bar{O})]}{\sqrt{\sum_{i=1}^{N}(P_i - \bar{P})^2 \times \sum_{i=1}^{N}(O_i - \bar{O})^2}} \quad (8)$$

Where $\boldsymbol{P_i}$ and $\boldsymbol{O_i}$ are the predicted and observed pollutant concentrations, respectively. *N* is the

total number of the predictions used for comparisons, and $\overline{\boldsymbol{P}}$ and $\overline{\boldsymbol{O}}$ represents the average of the prediction and observation, respectively.

Model Validation

<u>Air pollutants simulations in the NCP.</u> Fig. S2 shows the temporal profiles of observed and calculated near-surface $PM_{2.5}$, O_3, NO_2, SO_2 and CO concentrations averaged over monitoring sites in the NCP from 05 December 2015 to 06 March 2016. The model generally tracks well the diurnal variation of near-surface $PM_{2.5}$ concentrations ([$PM_{2.5}$]) in the NCP, with an *IOA* of 0.92, but slightly underestimates [$PM_{2.5}$], with a *MB* of -1.2 μg m^{-3}. The model successfully reproduces the temporal variations of near-surface O_3 concentrations compared to observations in the NCP, e.g., peak O_3 concentrations in the afternoon due to active photochemistry and low O_3 concentrations during nighttime caused by the NO_x titration, with an *IOA* of 0.91. However, the model generally overestimates the O_3 concentration during nighttime, with a *MB* of 0.1 μg m^{-3}. The model also reasonably well yields the NO_2 diurnal profiles with peaks in the evening, with an *IOA* of 0.88 and a *MB* of 1.6 μg m^{-3}, but sometimes there are considerable overestimations and underestimations. The model generally performs reasonably well in predicting the temporal variation of SO_2 concentrations against measurements, with an *IOA* of 0.88. However, considering that SO_2 is mainly emitted from point sources and its simulations are more sensitive to the wind field uncertainties (*33*), with the *RMSE* of -0.5 μg m^{-3}. Compared with measurements, the temporal profile of the near-surface CO concentration in the NCP is well simulated, with the *IOA* and *MB* of 0.95 and 0.0 mg m^{-3}, respectively.

<u>Spatial simulations of air pollutants in Eastern China.</u> Fig. S3 presents the spatial pattern of calculated and observed average near-surface concentrations of $PM_{2.5}$, O_3, NO_2, and SO_2 along with simulated winds from 05 December 2015 to 06 March 2016 in Eastern China. In

general, the simulated air pollutants distributions are in good agreement with the measurements, but model biases still exist. The simulated winds are weak or calm during the simulation period, facilitating accumulation of air pollutants and causing the serious air pollution in Eastern China. The NCP is the most polluted region in Eastern China due to its massive air pollutants emissions, with the average near-surface [$PM_{2.5}$] generally exceeding 75 μg m^{-3}. The highest average near-surface [$PM_{2.5}$] of more than 115 μg m^{-3} are observed in Hebei, Henan, and Shandong, which are well reproduced by the model. The simulated O_3 concentrations are rather low in the NCP, ranging from 20 to 60 μg m^{-3}, consistent with measurements. The low O_3 concentration during wintertime haze episodes in the NCP is primarily caused by the weak insolation further attenuated by clouds and aerosols, the titration of high NO_x emissions, and lack of the O_3 transport from outside (*34*). Although significant effort has been made to mitigate air pollutants emissions in the NCP, the observed and simulated average NO_2 and SO_2 concentrations are still high, varying from 2 to 70 μg m^{-3} and 5 to 100 μg m^{-3}, respectively. Interestingly, the simulated high SO_2 concentrations are mainly concentrated in cities and their surrounding areas, but the uniform distribution of NO_2 concentrations is predicted in the NCP, showing the substantial contribution of area sources.

Aerosol species simulations in Beijing. Fig. S4-6 provides the hourly variations of simulated and observed aerosol species at NCNST in Beijing from 05 December 2015 to 06 March 2016. Generally, the WRF-Chem model predicts reasonably the temporal variations of the aerosol species against the measurements. The WRF-Chem model yields the main peaks of the BC concentration compared to observations in Beijing, but frequently underestimates or overestimates the BC concentration, with *IOA*s of 0.72, 0.69, and 0.75, respectively. The BC level in Beijing is influenced by local emissions and to a large extent trans-boundary transport from outside during haze days, so its simulation is sensitive to uncertainties from emissions

and meteorological fields (*35, 36*). The model performs well in simulating the hourly variation of OA concentrations, with *IOA*s of 0.72, 0.64, and 0.78, respectively, in the three separated stages during the study episodes. However, the model still has difficulties in simulating the OA concentrations, with the *MBs* of -6.9, -2.5, and 1.9 μg m^{-3}, respectively. As a primary species, primary organic aerosols (POA) are also more sensitive to emissions and meteorological fields. As for secondary organic aerosols (SOA), its formation and transformation mechanism in the atmosphere still remains elusive. Many factors have potentials to influence the SOA simulation, such as meteorology, measurements, precursors emissions, and SOA treatments (*4*). The model tracks reasonably the temporal variation of the observed sulfate concentrations, and *IOA*s higher than 0.75, except the episode from 05 January 2015 to 04 February 2016. Aside from SO_2 emissions and simulated meteorological fields, the SO_2 oxidation mechanism in the atmosphere also plays an important role in the sulfate simulation. In addition to direct emissions and SO_2 gas-phase oxidations by hydroxyl radicals (OH) and stabilized criegee intermediates (sCI), the SO_2 oxidation in aerosol water by O_2 catalyzed by Fe^{3+} is considered (*37*). Recent studies have proposed that the aqueous oxidation of SO_2 by NO_2 under the condition of high relative humidity (RH) and NH_3 neutralization could interpret the efficient sulfate formation during wintertime haze events (*38, 39*). However, the mechanism is still not included in this study, which might further improve the sulfate simulation. The model also performs well in simulating the nitrate and ammonium concentrations against observations in Beijing, with *IOAs* generally higher than 0.70.

<u>Meteorological simulations in the NCP.</u> Fig. S7-13 show the diurnal profiles of observed and simulated T2, RH, WS10, and WD at seven typical monitoring sites in the NCP from 05 December 2015 to 06 March 2016. The WRF-Chem model performs well in reproducing the diurnal variability of surface temperature, especially in Tianjin, Nanjing, and Zhengzhou, with

*IOA*s higher than 0.85. The model has difficulty in reproducing the temporal variation of temperature in Beijing and Hefei, with *IOA*s of 0.63 and 0.66. The model has a good performance in simulating the variation of RH, with *IOA*s generally higher than 0.75. There exists overestimation in Beijing, Shijiazhuang, Zhengzhou, Jinan, and Hefei, with *MBs* ranging from 0.8% to 8.1%. The model also has a difficulty in simulating the wind fields. Generally, the WRF-Chem model performs well in reproducing the diurnal variability of wind speed, but the overestimation exists, except Tianjin site, with *MBs* varying from 0.13 to 0.24 m s^{-1}. The WD is also well simulated in Tianjin, Hefei, Zhengzhou, and Nanjing, with *IOA*s higher than 0.80, but the model has difficulty in reproduce the WD variation in Beijing and Shijiazhuang, with *IOA*s of 0.51 and 0.45.

In general, the model performs well in simulating the meteorological conditions in the NCP.

Downward solar radiation simulations in the NCP. Fig. S14 presents the daily profiles of simulated and observed SWDOWN at the ground surface in Beijing, Jiaozhouwan, Luancheng, Yuancheng, and Fengqiu from 05 December 2015 to 06 March 2016. The WRF-Chem model simulates well the daily variation of SWDOWN, especially in Fengqiu, Jiaozhouwan, Luancheng, and Yucheng, with *IOAs* higher than 0.75. The model is subject to overestimating the SWDOWN against measurements, with *MB*s ranging from 4.6 to 73.9 W m^{-2}. The SWDOWN reaching the ground surface is very sensitive to the cloud cover and optical thickness. However, the WRF-Chem model still has difficulties in accurately predicting the cloud cover and optical thickness, which might constitute one of the most important reasons for model biases of the SWDOWN. In addition, the horizontal resolution used in simulations cannot adequately resolve the cumulus clouds, also causing uncertainties in the simulations of the SWDOWN.

Ultraviolet (UV) radiation simulations in the NCP. Fig. S15 presents comparison of

measured and predicted diurnal profiles of ultraviolet (UV) radiation (wavelength at 200~420 nm) reaching the surface (UVDOWN) at four observation sites in the NCP during the episode. The simulated temporal variation of UVDOWN in F_{BASE} is generally consistent with the measurements, with IOAs close to or exceeding 0.80, but the model overestimation of UVDOWN is discernable, with *MBs* of 2.1, 2.1, 1.6, 2.3, and 1.4 W m^{-2} in Beijing, Fengqiu, Jiaozhouwan, Luancheng, and Yucheng, respectively, possibly caused by uncertainties in cloud simulations. However, in F_{Abs0}, the WRF-Chem model markedly overestimates UVDOWN against measurements, with *MBs* of about 3.0~5.0 W m^{-2} higher than those in the F_{BASE}.

Aerosol radiative properties simulations in the NCP. Aerosol radiative forcing mainly depends on AOD, SSA, and asymmetry parameter. The model validations of AOD and SSA are provided in this study to further evaluate the aerosol radiative effect on the air pollution. The OMI-retrieved AOD in the NCP during the study episode is compared with the simulation (Fig. S15). The average simulated and retrieved AOD during the simulation period is 0.48 and 0.52, respectively, with *MB* of -0.04. Fig.S16 shows the pattern comparison of the retrieved and simulated AOD on the selected day during the simulation period. The model reasonably reproduces the AOD distribution compared to the OMI retrieval in the NCP, but sometimes underestimates the AOD. It is worth noting that the simulated AOD is not only dependent on the column aerosol content and constituent, but also significantly influenced by RH controlling the aerosol hydroscopic growth. Additionally, the satellite retrieved AOD is subject to contamination by existence of clouds, and considering the high occurrence frequency of clouds during haze days, the retrieved AOD is generally higher than the simulation (*40-42*).

Aerosols are the mixture of absorbing and scattering constituents in the atmosphere. Their radiative effect of cooling or warming the atmosphere relies on many parameters, and SSA is one of the most important parameters (*43*). The hourly SSA observation was completed on 31 December 2015 at NCNST in Beijing, so we only take a comparison of hourly observed and

simulated SSA from 05 December 2015 to 31 December 2015. Fig. S17 depicts the comparison of the measured and simulated diurnal profiles of SSA at 520nm at NCNST in Beijing during this study. The model performs reasonably in simulating the daily variation of SSA in Beijing, with an *IOA* of 0.71 and a *MB* of 0.0, but the overestimation or underestimation is rather large. SSA is the ratio of aerosol scattering to extinction, which is highly sensitive to the relative distribution of scattering and absorbing aerosol constituents in the atmosphere, and the RH determining the hygroscopic growth of aerosols. Therefore, the uncertainties of the simulated SSA probably originate from the model biases of aerosol constituents and the RH.

PBLH simulations in Beijing. Fig. S18 shows the temporal variations of the observed and simulated PBLH at a meteorological site in Beijing from 05 December 2015 to 31 December 2015. The PBLH observation campaign also finished at the end of 2015. The average PBLH at 12:00 Beijing Time (BJT) during the episode at the meteorological site is 465.2 m, with the minimum of 101.8 m and the maximum of 1017.9 m, showing decreased PBLH during the haze episode. In general, the WRF-Chem model tracks reasonably the daily variation of the PBLH in Beijing, with an *IOA* of 0.69. However, the model has difficulties in reproducing the observed very low PBLH, e.g., less than 200 m. The PBLH varies substantially with time due to many factors including large-scale dynamics, cloudiness, convective mixing, and the diurnal cycle of solar radiation (*44*). Therefore, the simulation uncertainties of meteorological conditions constitute the main reason for the simulation bias of PBLH. For example, the overestimation of SWDOWN at 12:00 BJT (Fig. S10a) probably causes the overestimation of PBLH in Beijing.

In general, the simulated variations of SWDOWN, PBLH, aerosol radiative properties, air pollutants ($PM_{2.5}$, O_3, NO_2, SO_2, CO) and aerosol species are in good agreement with observations, indicating that the simulations of meteorological conditions, chemical processes and the emission inventory used in the WRF-Chem model are reasonable, providing a reliable

basis for the further study.

Cloud variations due to the AARI: The AARI effect not only alters the aerosol concentration and distribution in the PBL, also perturbs the temperature and wind field, which could influence clouds and radiation. Less surface heating and atmospheric stabilization both impact cloud dynamics by reducing vertical sensible and latent heat exchanges and modulating convective development and cloud formation, whereas warming in the atmosphere decreases relative humidity or supersaturation and may contribute to cloud dissipation (*45*). In addition, depending on the aerosol aging processes, the coupling between radiative and cloud-forming effects of light absorbing aerosols in the atmosphere may cancel out the overall effects of aerosols on clouds and precipitation (*46*), since the direct and indirect effects often operate in opposite directions. Fig. S33 presents the daily variation of the average daytime cloud optical depth (COT) and cloud fraction (CF) caused by the AARI effect over the NCP from 05 December 2015 to 06 March 2016. The AARI effect generally increases the COT and CF during the episode slightly, with an average enhancement of 2.4% and 1.4%, respectively. The COT and CF increase more during the nighttime than daytime, with the enhancement of 3.1% and 2.1%, respectively. The increased low-level clouds could intensify the AARI effect during the nighttime through the heating effect of cloud absorption of the longwave radiation from the surface.

Table S1. WRF-Chem model configurations

Regions	East Asia
Simulation period	December 05, 2015 - March 06, 2016
Domain size	600 × 600
Domain center	35°N, 114°E
Horizontal resolution	9km × 9km
Vertical resolution	35 vertical levels with a stretched vertical grid with spacing ranging from 30 m near the surface, to 500 m at 2.5 km and 1 km above 14 km
Microphysics scheme	WSM 6-class graupel scheme (*15*)
Boundary layer scheme	MYJ TKE scheme (*16*)
Surface layer scheme	MYJ surface scheme (*16*)
Land-surface scheme	Unified Noah land-surface model (*17*)
Longwave radiation scheme	Goddard longwave scheme (*18*)
Shortwave radiation scheme	Goddard shortwave scheme (*19*)
Meteorological boundary and initial conditions	NCEP 1°×1° reanalysis data
Chemical initial and boundary conditions	MOZART 6-hour output (*20*)
Anthropogenic emission inventory	Developed by Zhang et al. (*21*) and Li et al. (*22*) with 2015 base year, and SAPRC-99 chemical mechanism
Biogenic emission inventory	MEGAN model developed by Guenther et al. (*23*)
Model spin-up time	100 hours

Table S2. Aerosol optical constants used in the calculation of aerosol optical properties.

Aerosols	Refractive Index (**λ**=380nm)	Refractive Index (**λ**=550nm)
Black carbon	1.75-0.75i	1.75-0.72i
Primary organic aerosol	1.55-0.14i	1.55-0.03i
Secondary organic aerosol	$1.55-2.0\times10^{-3}i$	$1.55-2.0\times10^{-3}i$
Sulfate	$1.44-1.0\times10^{-8}i$	$1.43-1.0\times10^{-8}i$
Nitrate	$1.44-1.0\times10^{-8}i$	$1.43-1.0\times10^{-8}i$
Ammonium	$1.44-1.0\times10^{-8}i$	$1.43-1.0\times10^{-8}i$
Water	$1.35-2.0\times10^{-9}i$	$1.34-2.5\times10^{-9}i$
Dust	$1.53-1.4\times10^{-2}i$	$1.53-5.5\times10^{-3}i$

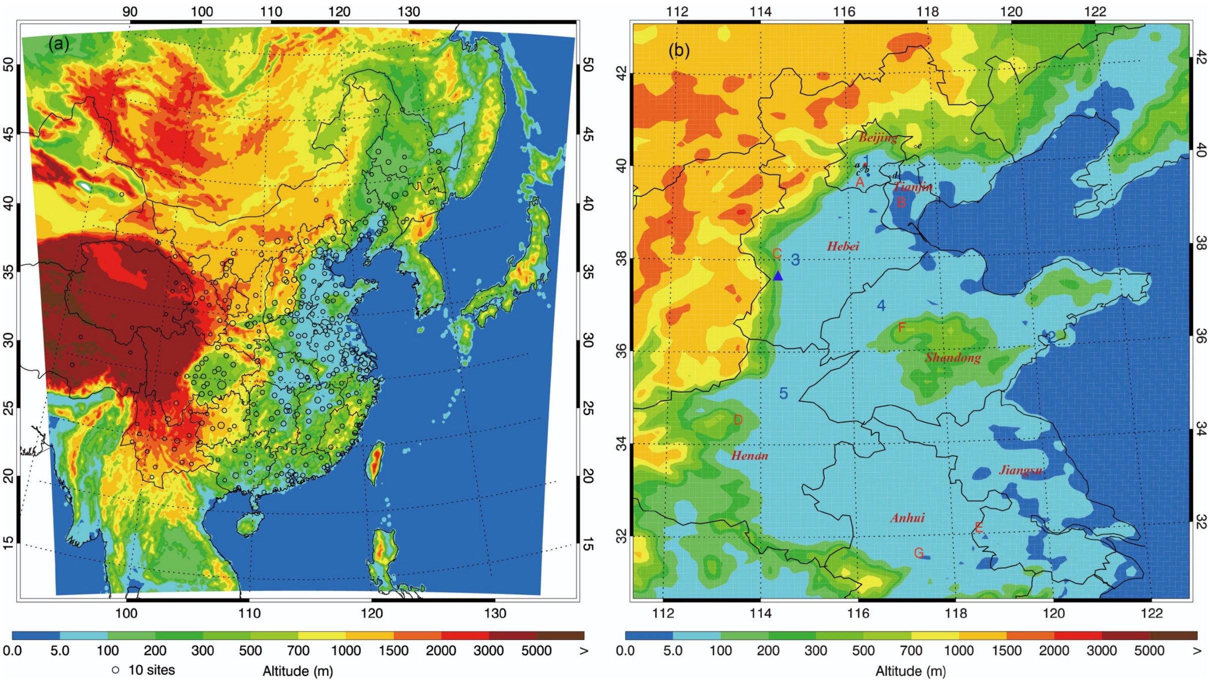

Fig. S1. (a) WRF-Chem simulation domain with topography and (b) NCP. In (a), the blue circles represent centers of cities with ambient monitoring sites and the size of blue circles denotes the number of ambient monitoring sites of cities. In (b), the black diamond with lowercase letters denotes the five SSA observation sites in the NCP. a: urban Beijing; b: CAMS site in Beijing; c: RADI site in Beijing; d: Xianghe site; e: Xinglong site. The red numbers denote the CERN sites with the solar radiation measurement. 1: Beijing; 2: Jiaozhouwan; 3: Luancheng; 4: Yucheng; 5: Fengqiu. The black capital letters denote the seven meteorological sites in the NCP. A: Beijing; B: Tianjin; C: Shijiazhuang; D: Zhengzhou; E: Nanjing; F: Jinan; G: Hefei. The blue filled circles indicate the NCNST site in Beijing, respectively, and the red filled rectangle denotes the meteorological site with PBLH in Beijing. The blue filled triangle shows the observation site with vertical profile in Shijiazhuang.

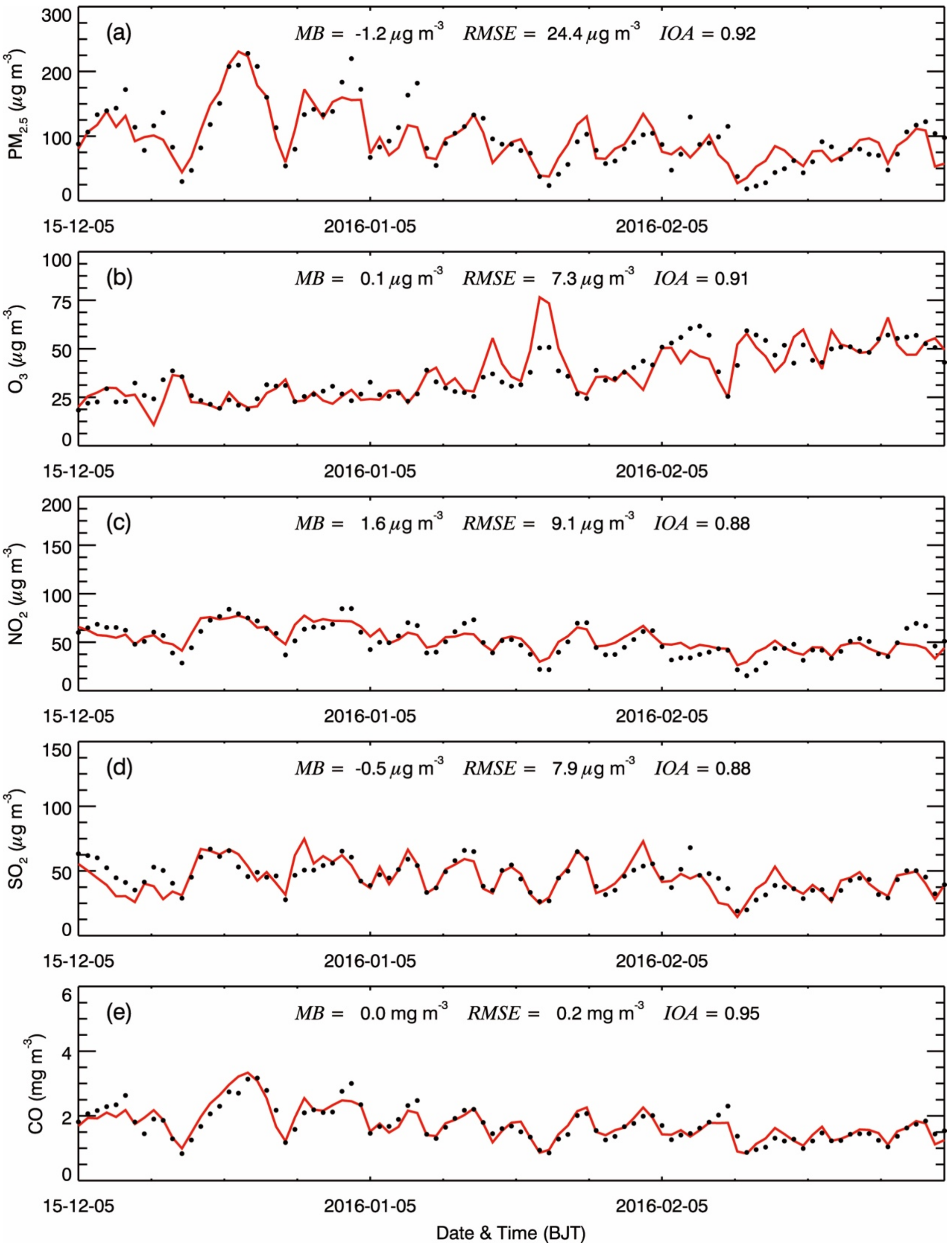

Fig. S2. Comparison of observed (black dots) and simulated (solid red lines) diurnal profiles of near-surface daily mass concentrations of (a) $PM_{2.5}$, (b) O_3, (c) NO_2, (d) SO_2, and (d) CO averaged at monitoring sites in the NCP from 05 December 2015 to 06 January 2016.

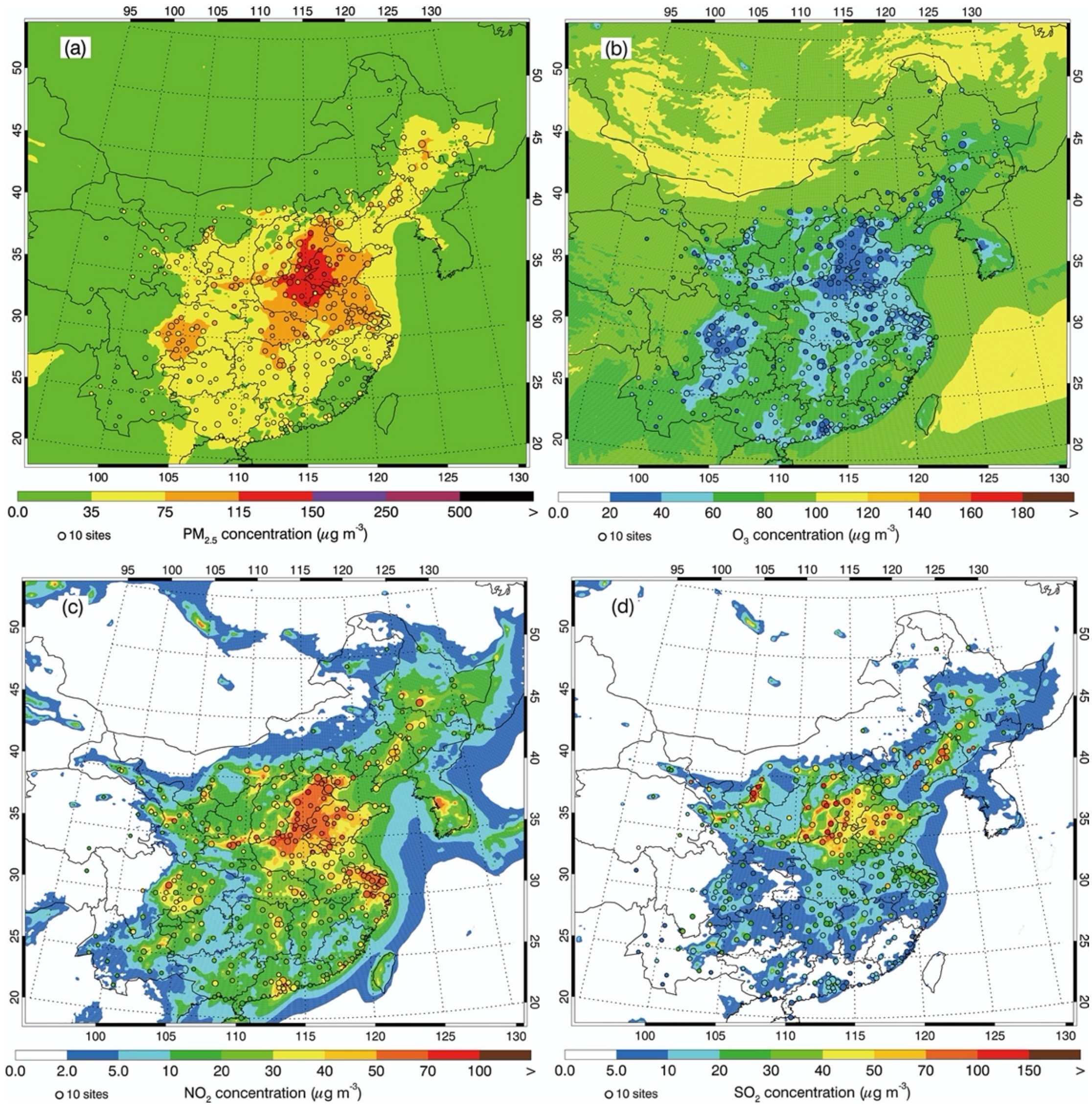

Fig. S3. Pattern comparisons of simulated (color counters) vs. observed (colored circles) near-surface mass concentrations of (a) $PM_{2.5}$, (b) O_3, (c) NO_2, and (d) SO_2 averaged from 05 December 2015 to 06 March 2016.

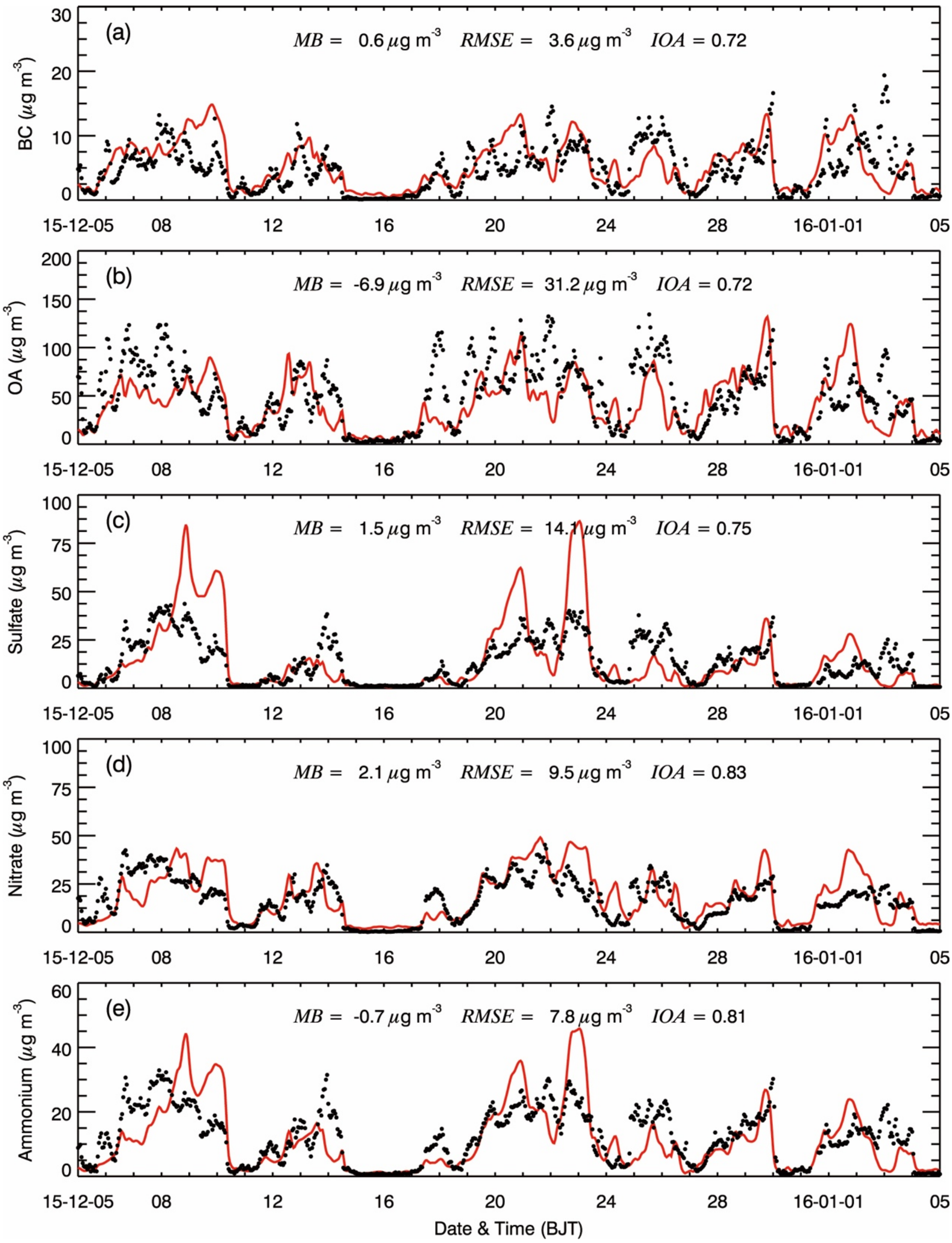

Fig. S4. Comparison of measured (black dots) and simulated (black line) diurnal profiles of submicron aerosol species of (a) BC, (b) OA, (c) sulfate, (d) nitrate, and (e) ammonium at NCNST site in Beijing from 05 December 2015 to 04 January 2016.

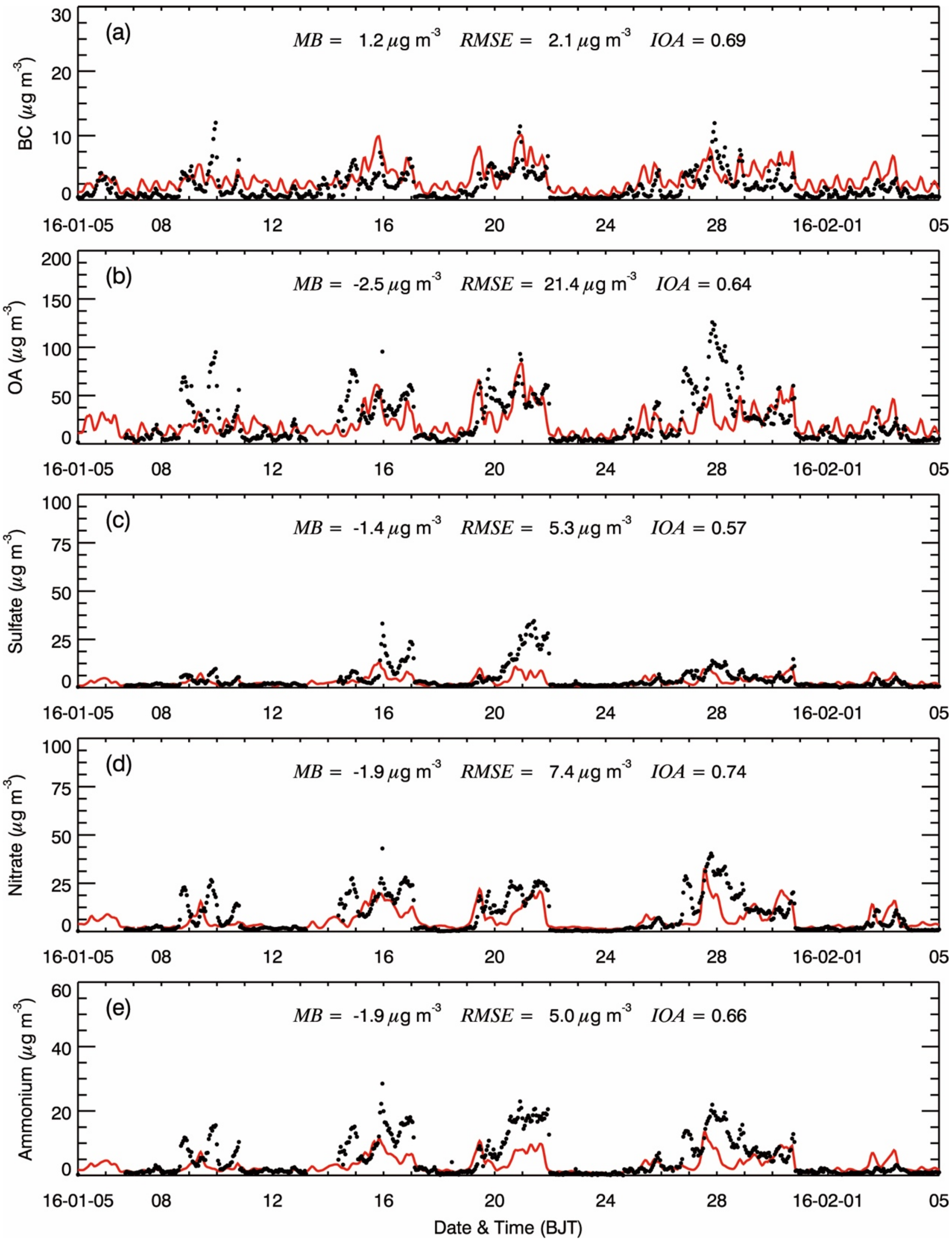

Fig. S5. Comparison of measured (black dots) and simulated (black line) diurnal profiles of submicron aerosol species of (a) BC, (b) OA, (c) sulfate, (d) nitrate, and (e) ammonium at NCNST site in Beijing from 05 January 2015 to 05 February 2016.

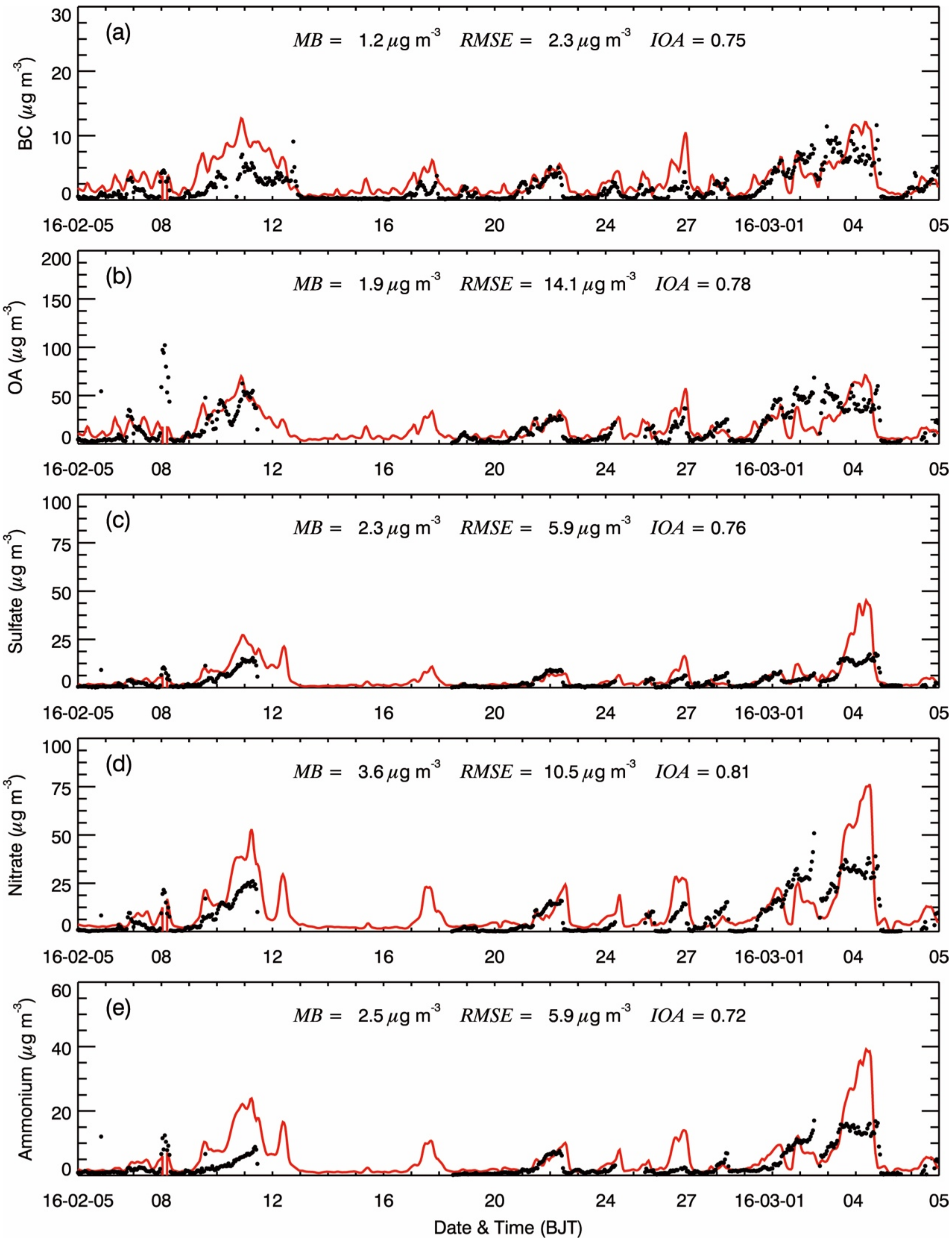

Fig. S6. Comparison of measured (black dots) and simulated (black line) diurnal profiles of submicron aerosol species of (a) BC, (b) OA, (c) sulfate, (d) nitrate, and (e) ammonium at NCNST site in Beijing from 05 February 2015 to 06 March 2016.

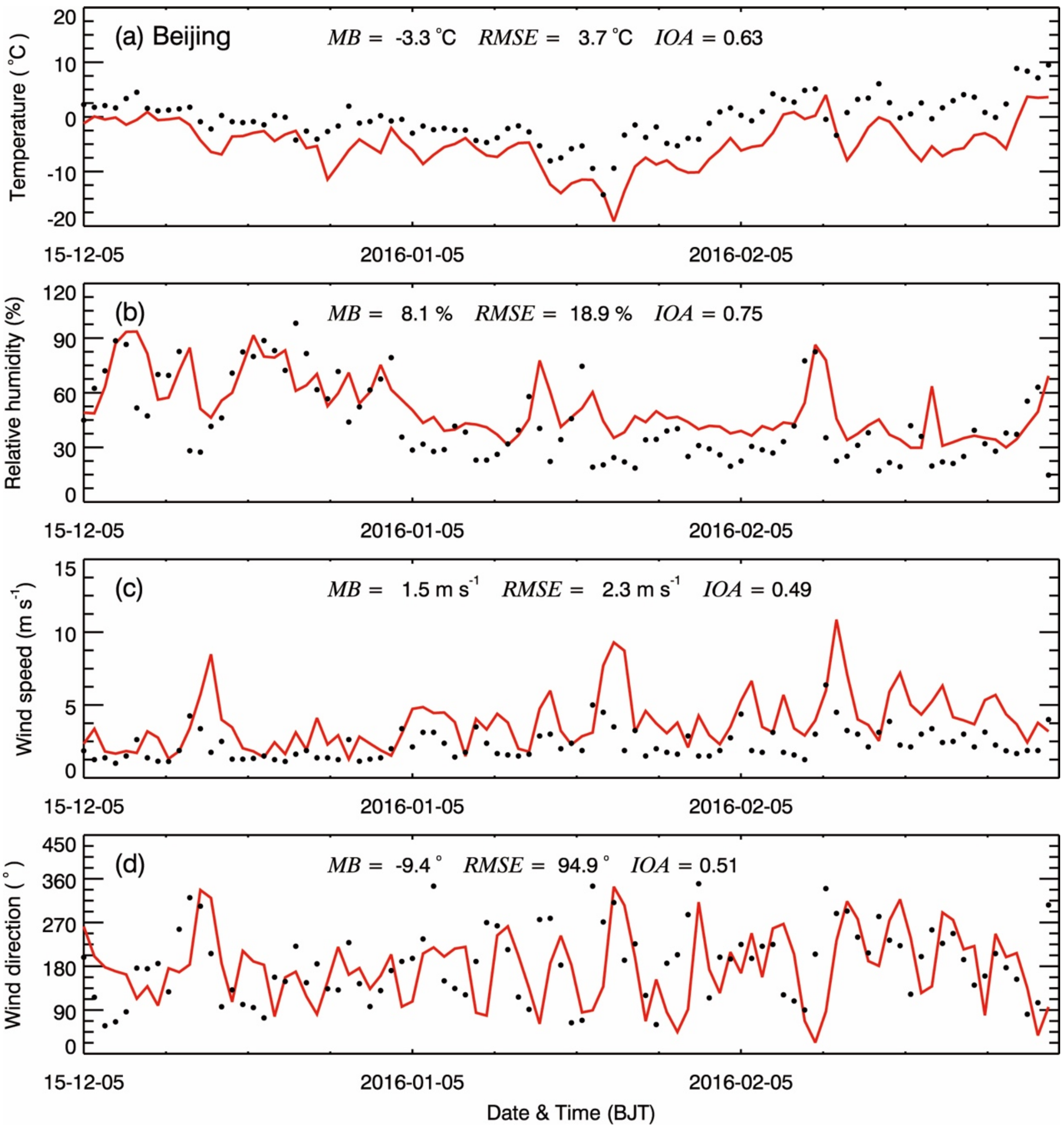

Fig. S7. Temporal variations of simulated (red lines) and observed (black dots) near-surface daily temperature (T), relative humid (RH), wind speed at 10m (WS10) and wind direction (WD) at Beijing meteorological monitoring site from 05 December 2015 to 06 March 2016.

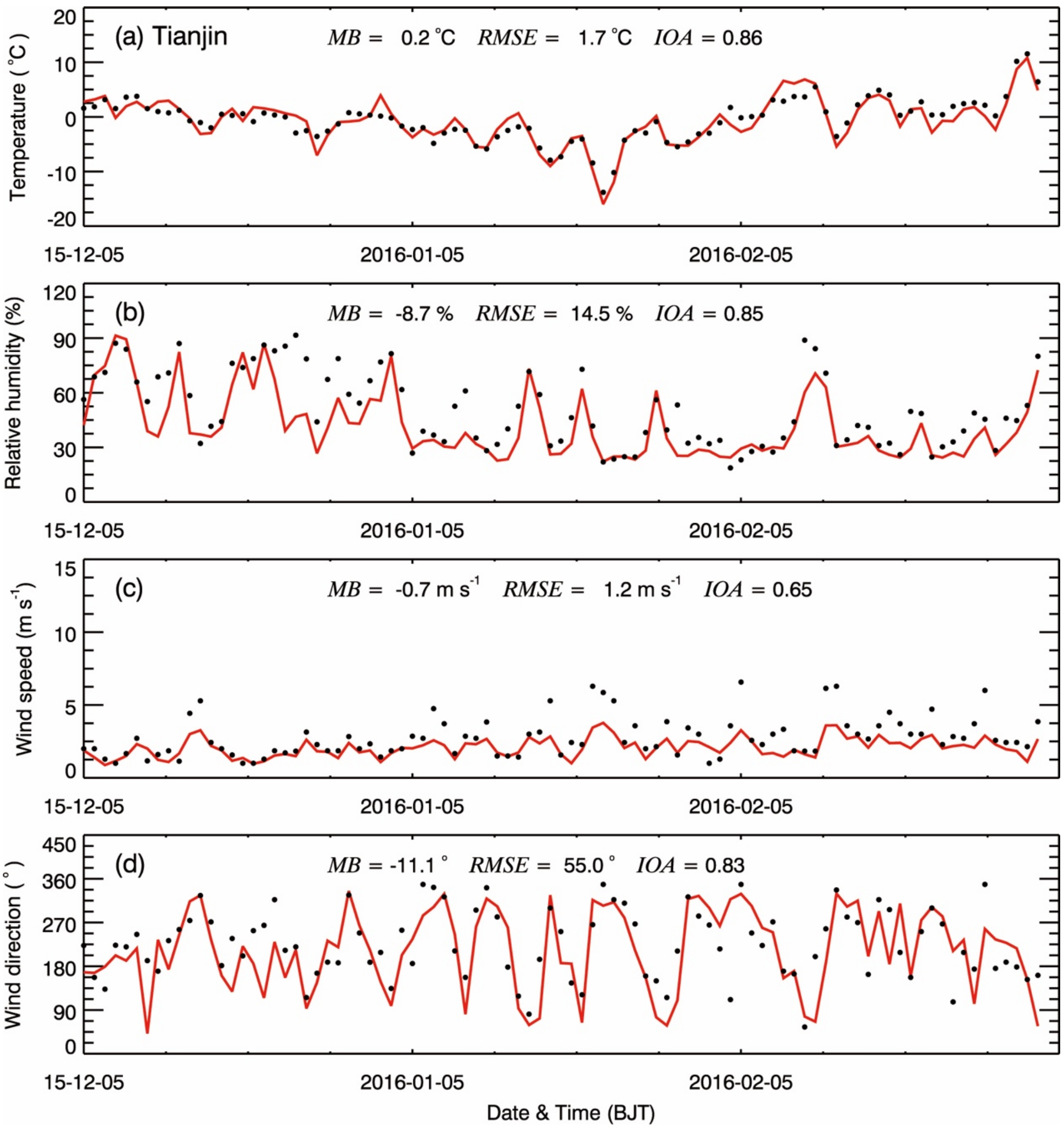

Fig. S8. Same as Fig. S7, but for Tianjin.

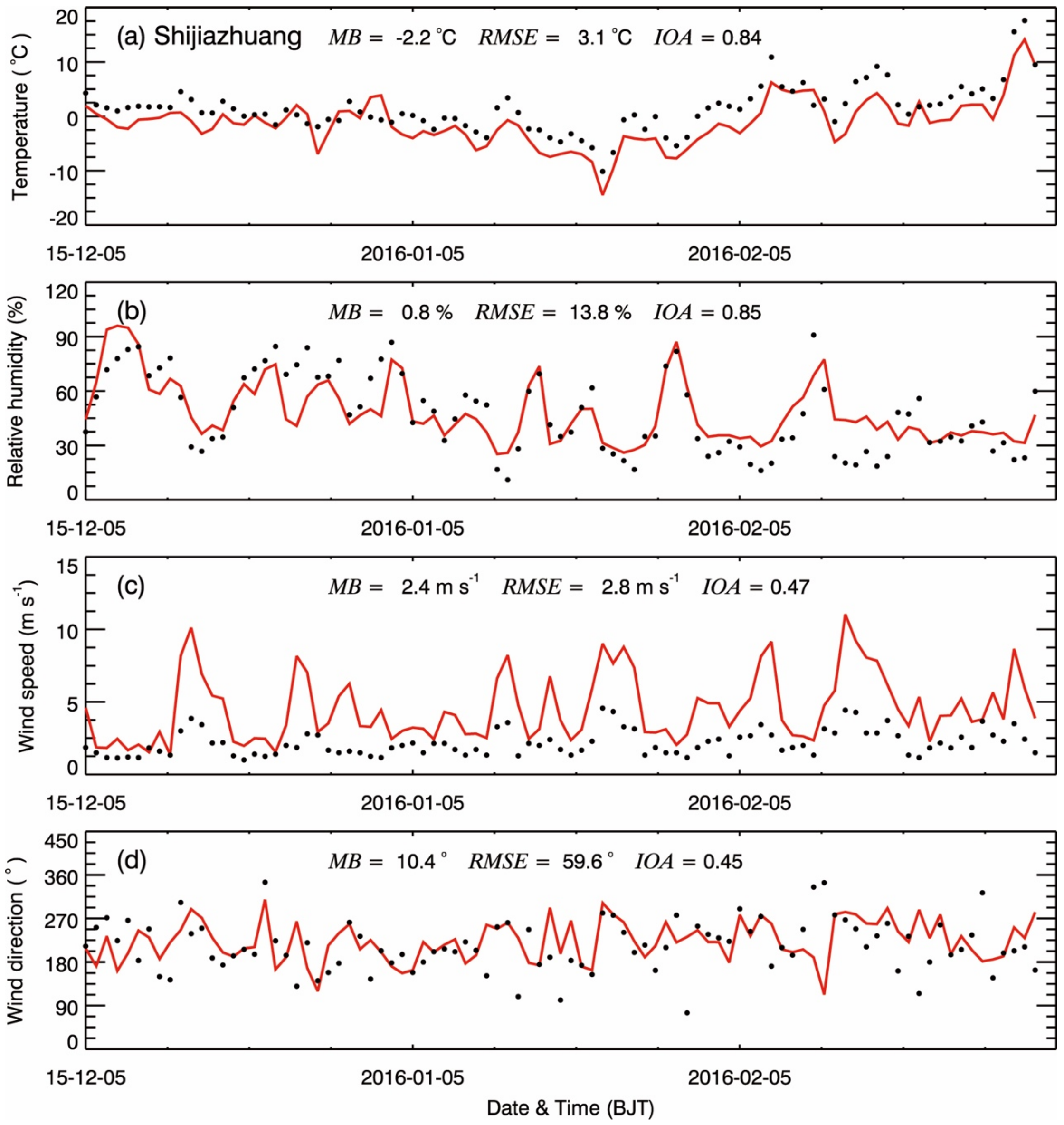

Fig. S9. Same as Fig. S7, but for Shijiazhuang.

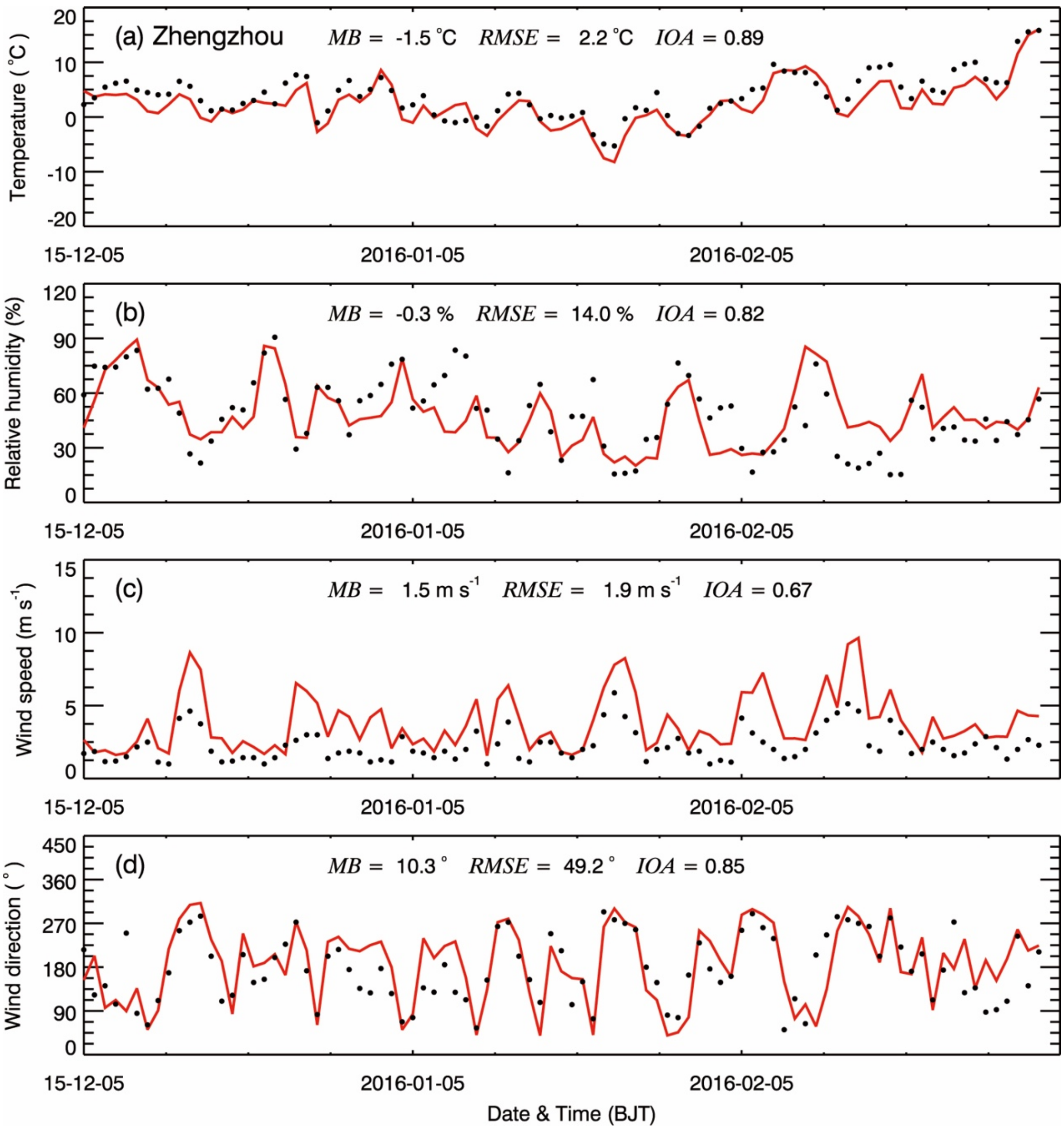

Fig. S10. Same as Fig. S7, but for Zhengzhou.

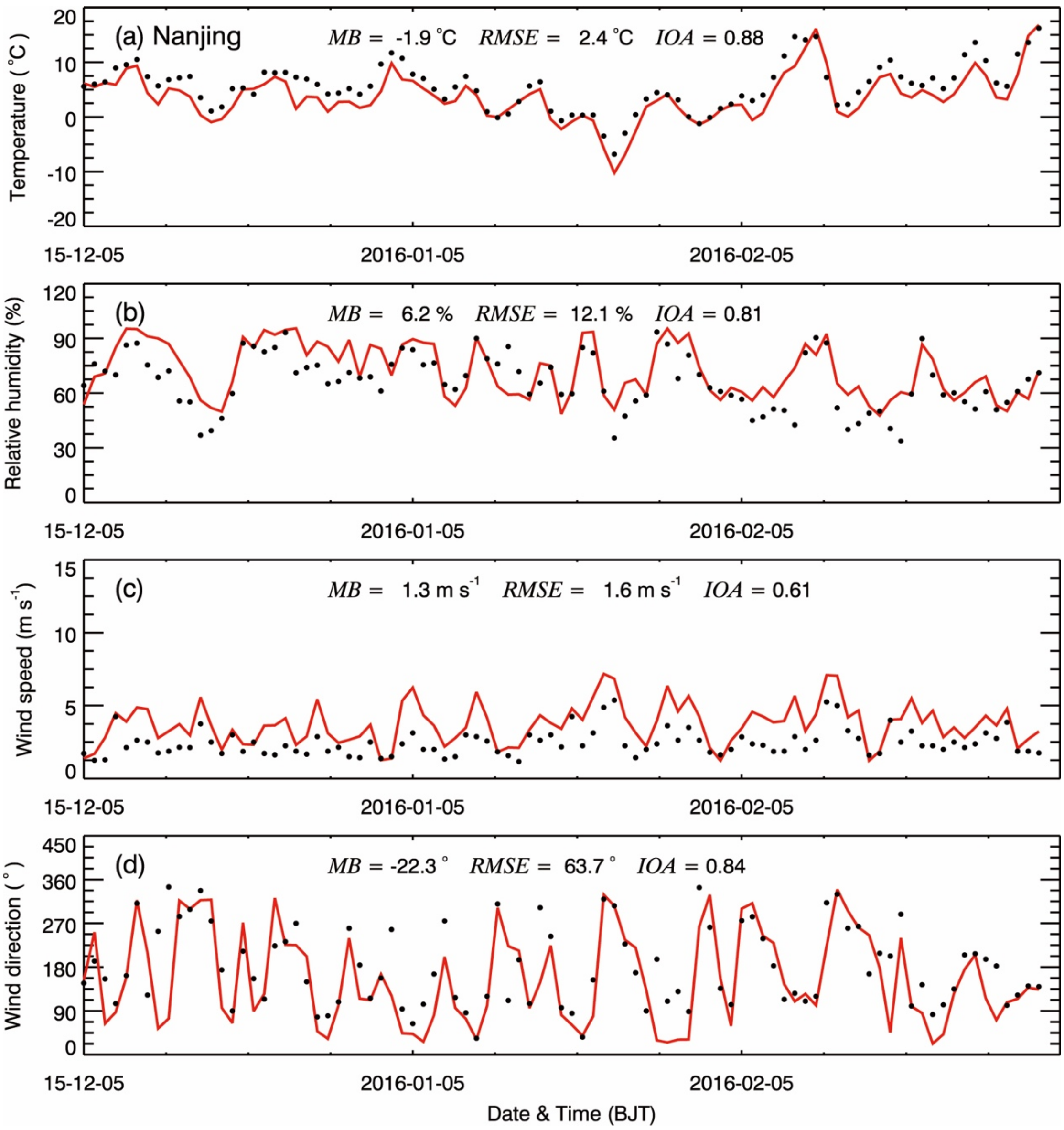

Fig. S11. Same as Fig. S7, but for Nanjing.

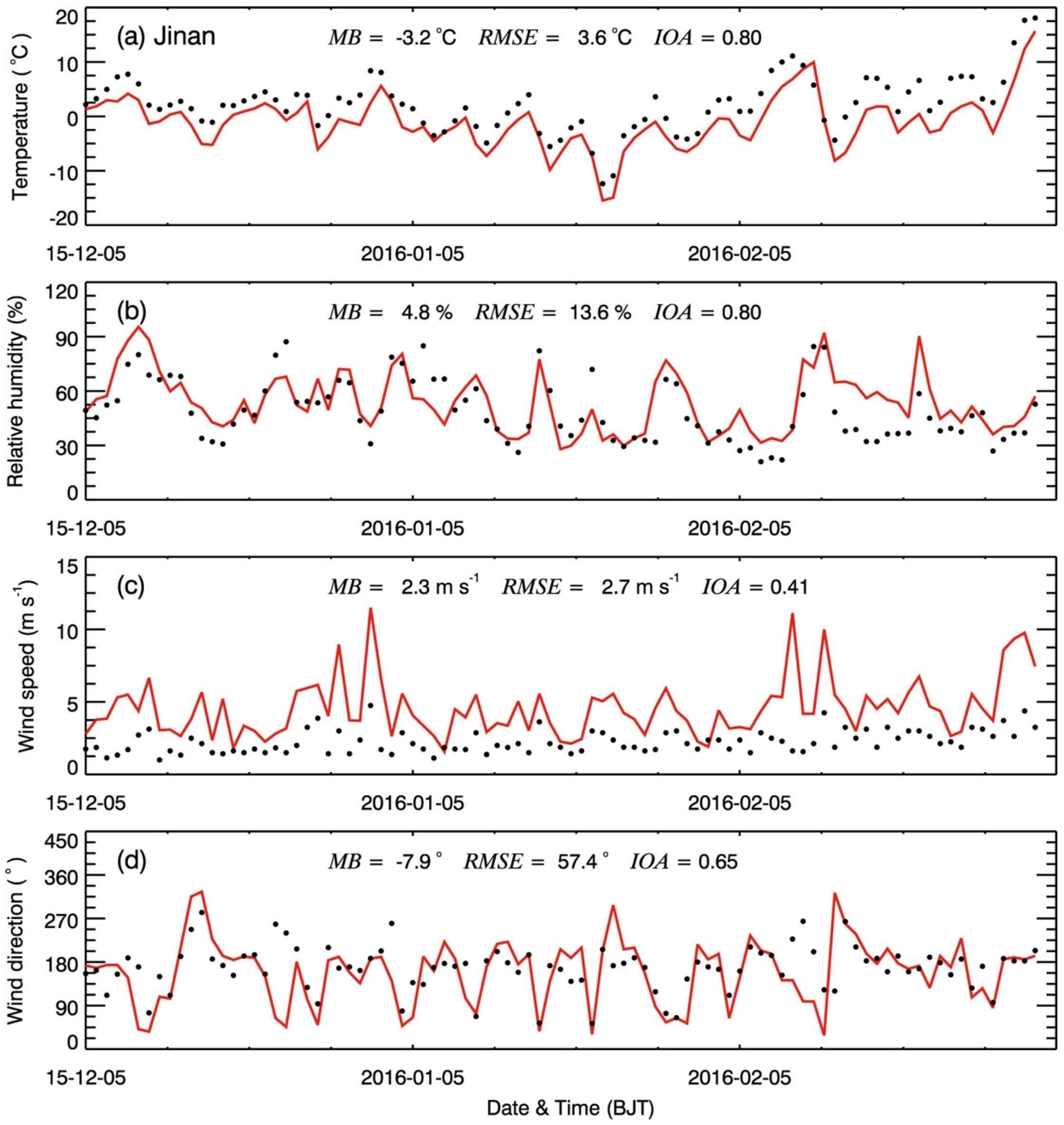

Fig. S12. Same as Fig. S7, but for Jinan.

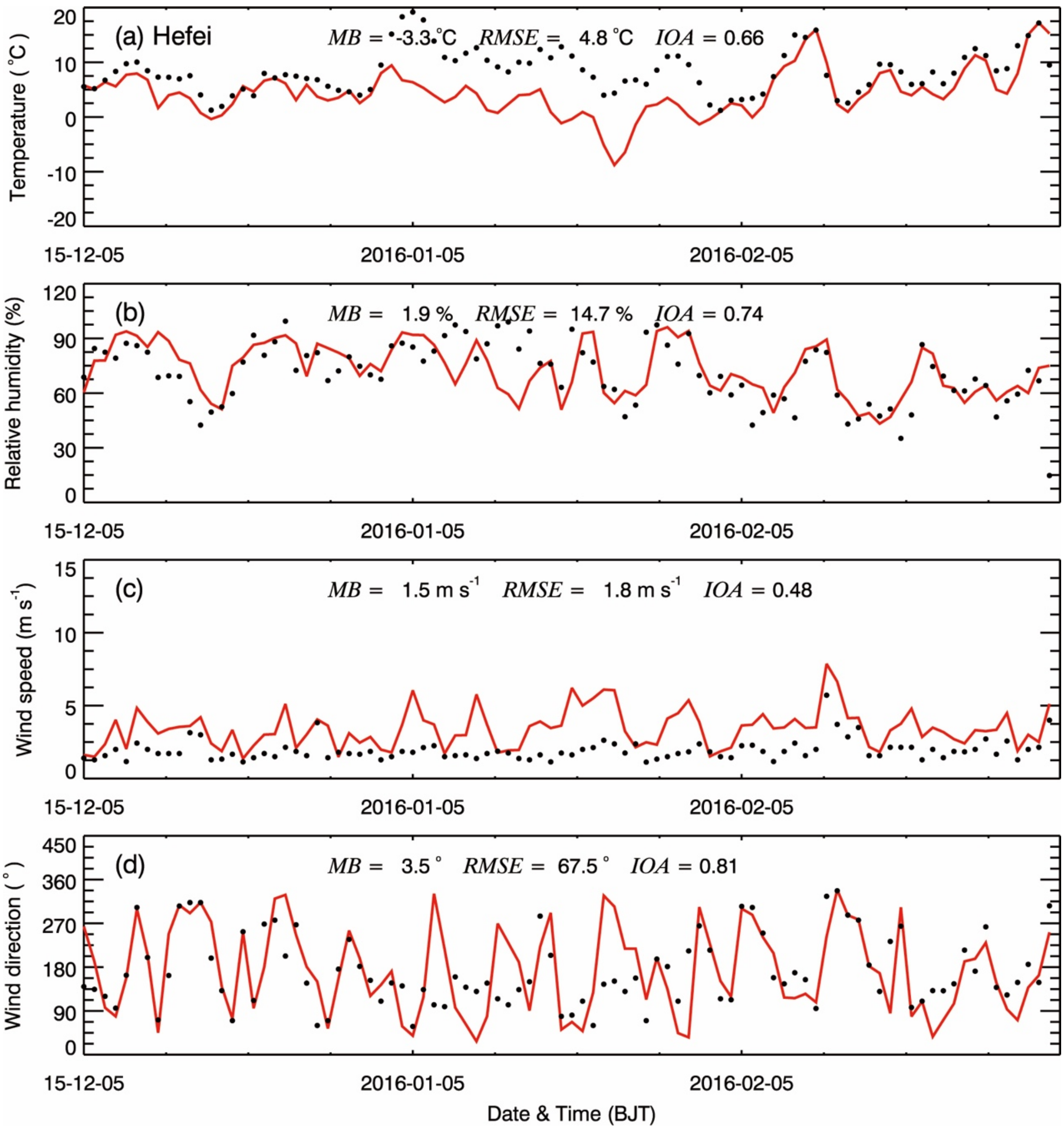

Fig. S13. Same as Fig. S7, but for Hefei.

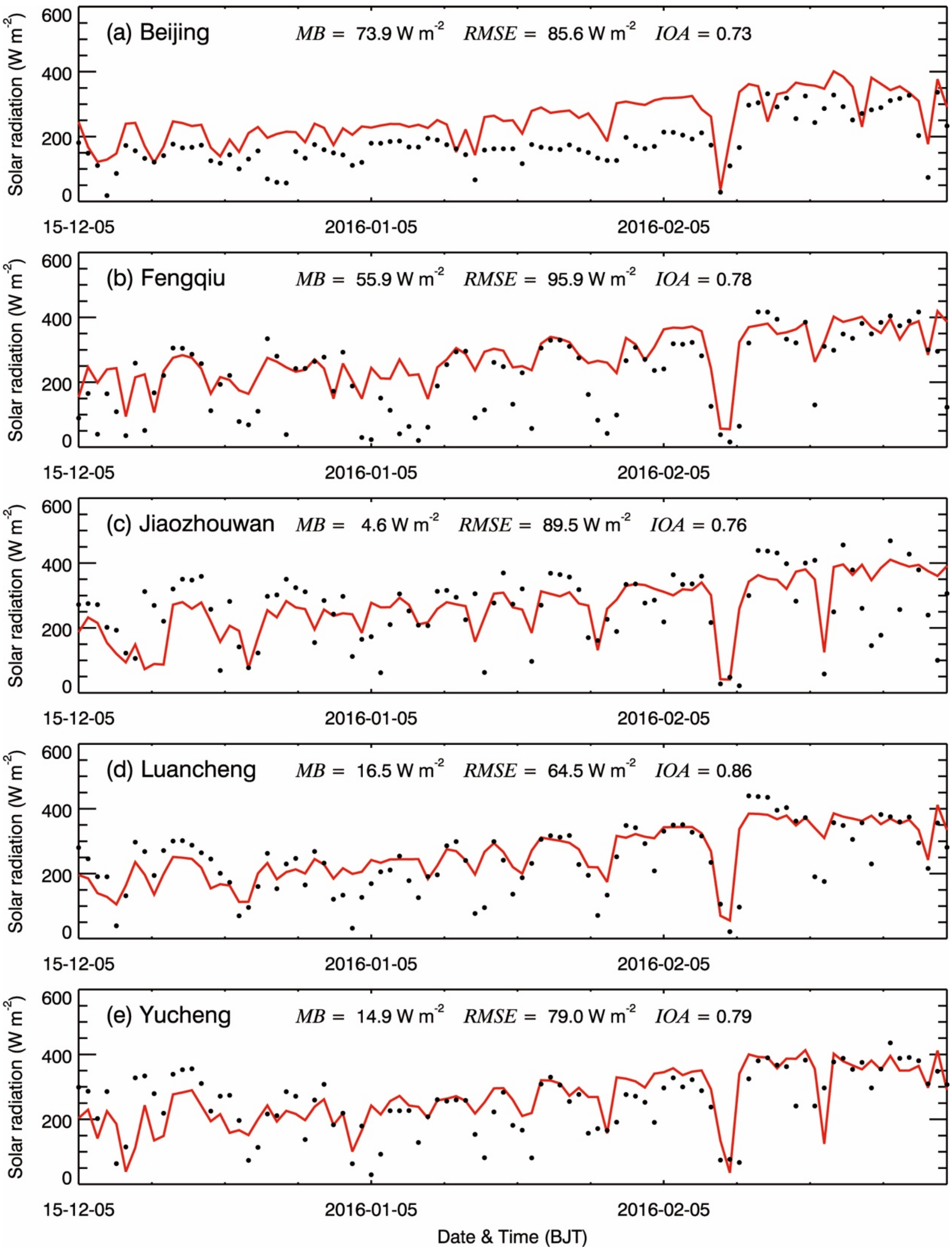

Fig. S14. Comparison of measured (black dots) and predicted (red line) daily profiles of the SWDOWN reaching the ground surface in (a) Beijing, (b) Fengqiu, (c) Jiaozhouwan, (d) Luancheng, and (e) Yucheng from 05 December 2015 to 06 March 2016.

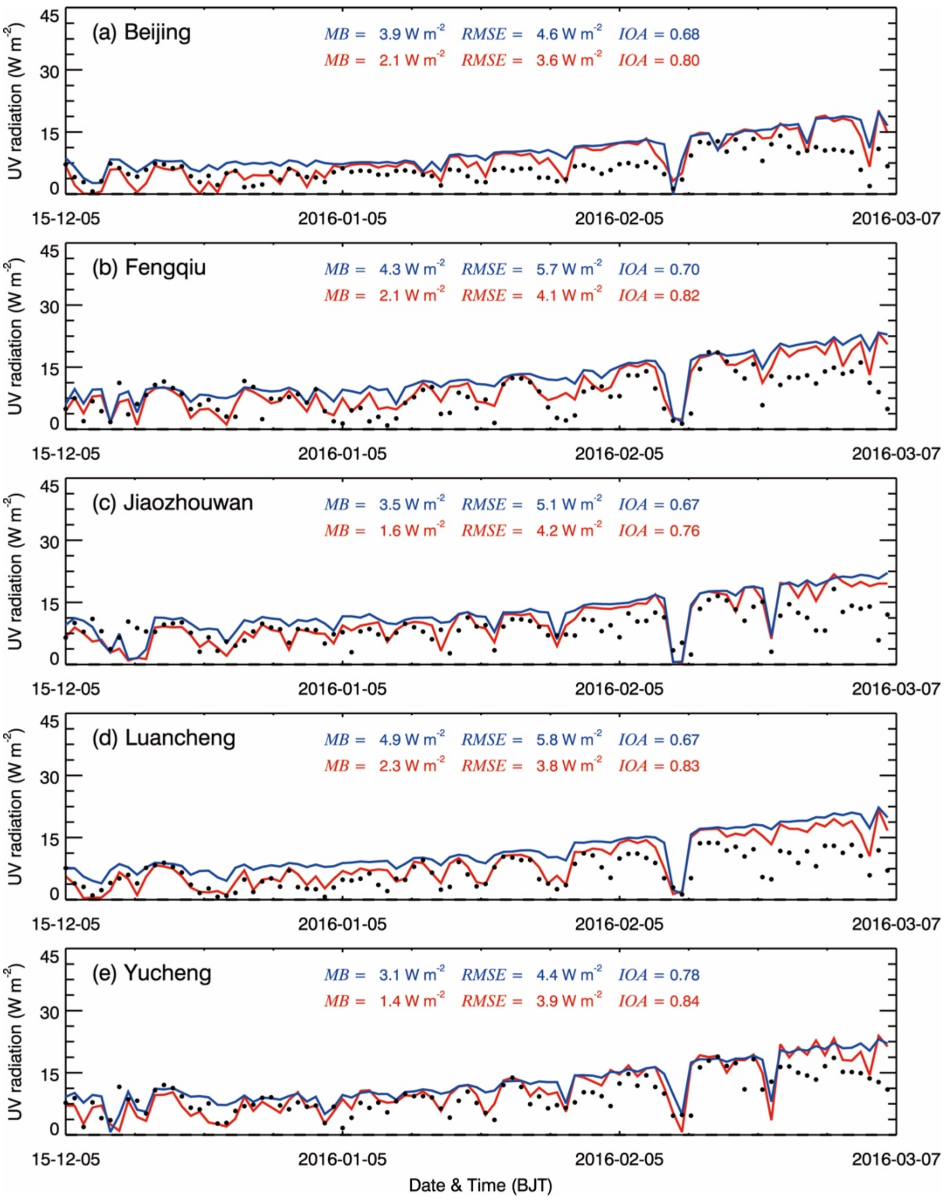

Fig. S15. Comparison of measured (black dots) and predicted daily profiles of the UVDOWN in F_{BASE} (red line) and F_{Abs0} (blue line) reaching the ground surface in (a) Beijing, (b) Fengqiu, (c) Jiaozhouwan, (d) Luancheng, and (e) Yucheng from 05 December 2015 to 06 March 2016.

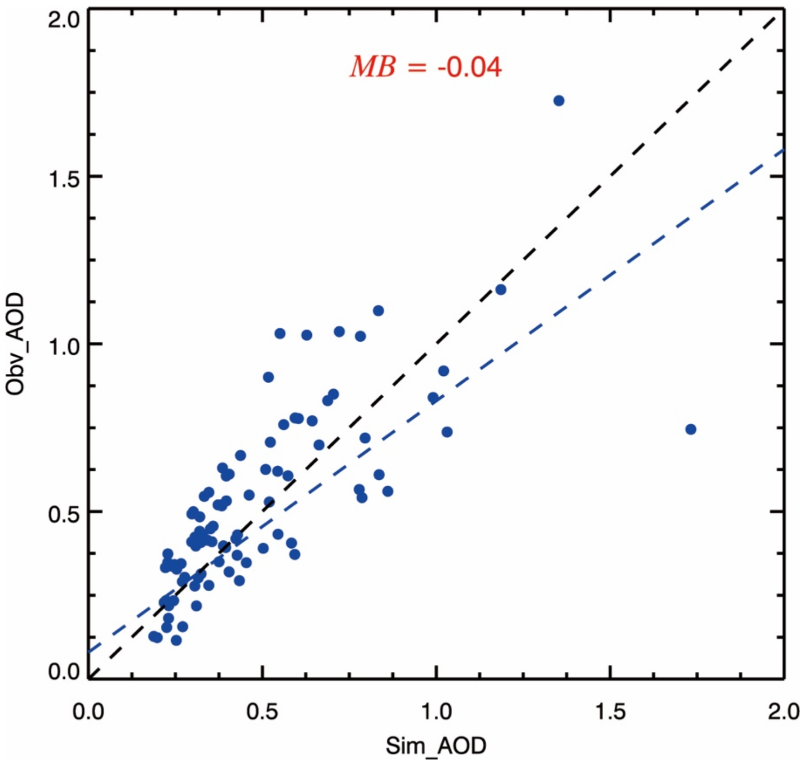

Fig. S16. Scatter plot of the OMI daily retrieved and simulated AOD averaged in the NCP from 05 December 2015 to 06 March 2016.

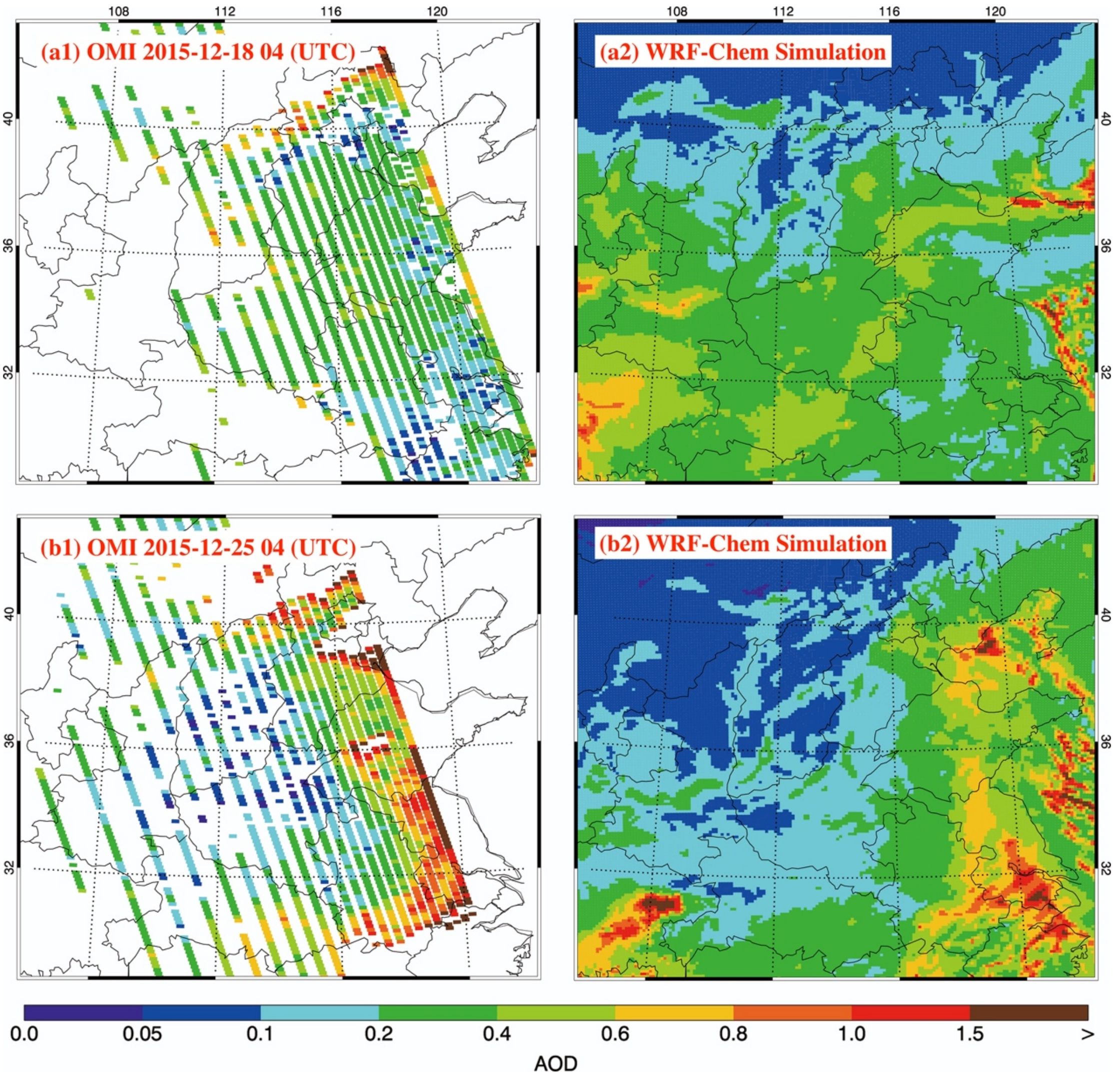

Fig. S17. Pattern comparison of the OMI retrieved and simulated AOD on the selected day during the study episode 05 December 2015 to 06 March 2016.

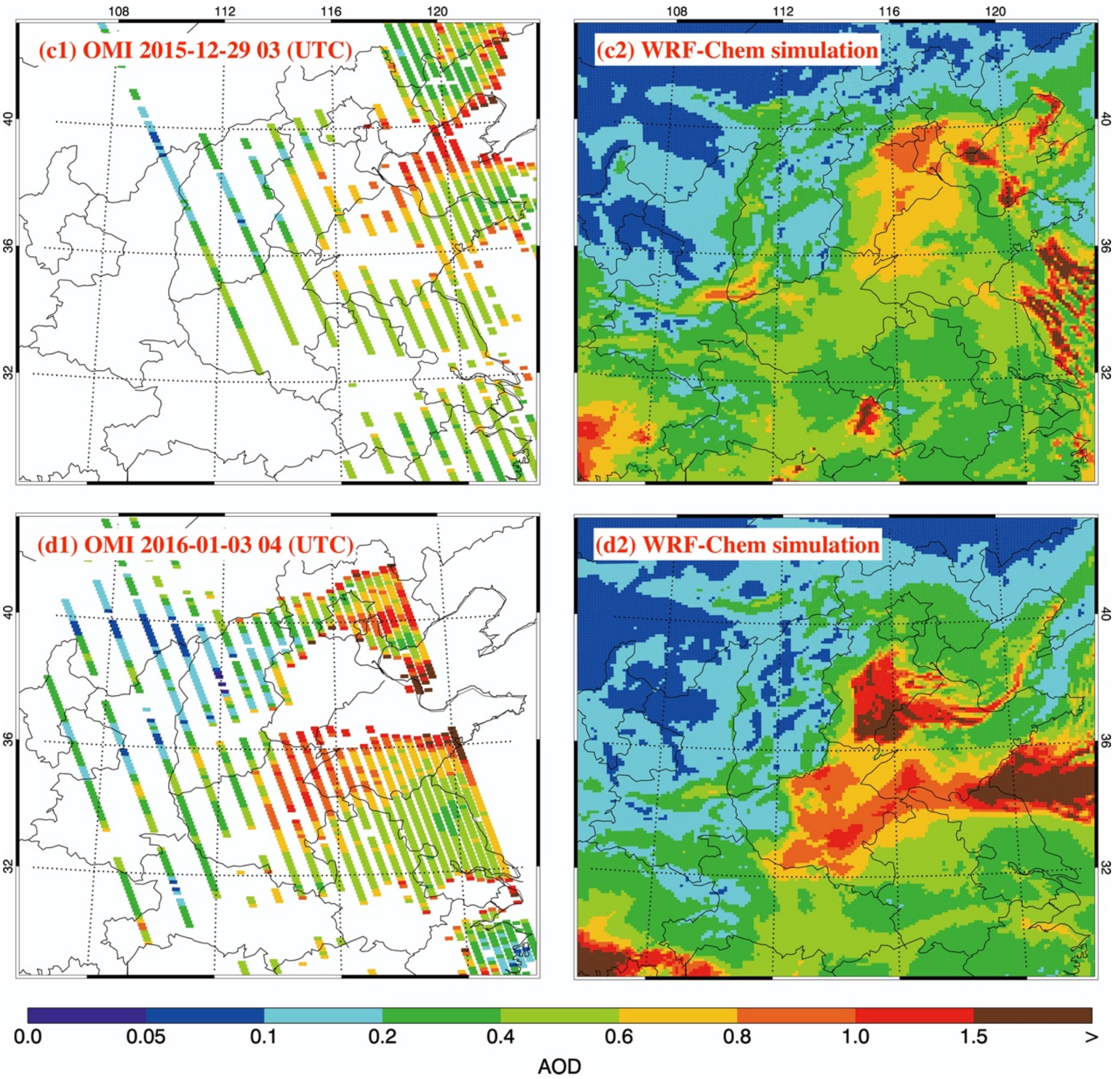

Fig. S17 Continued.

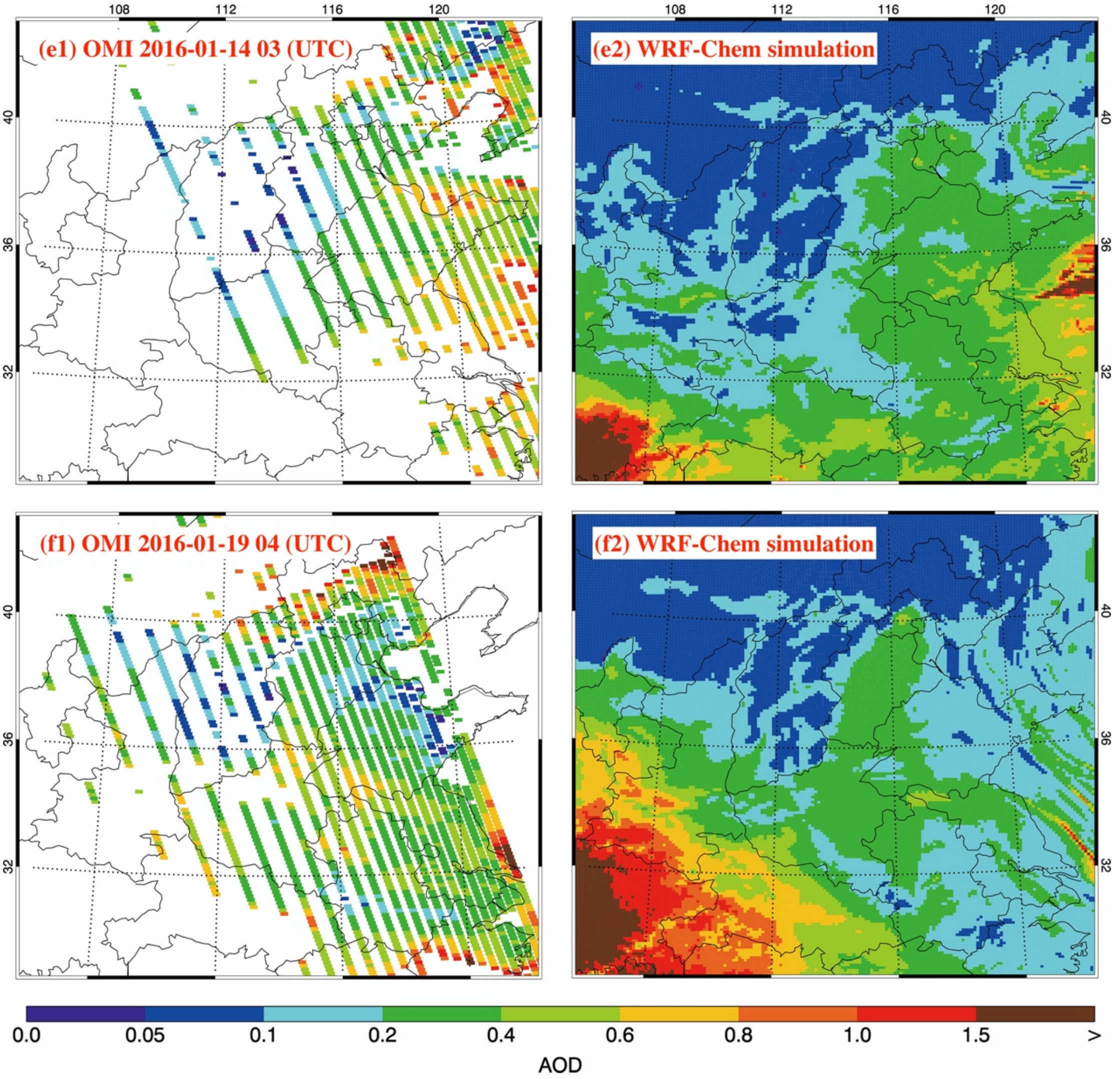

Fig. S17 Continued.

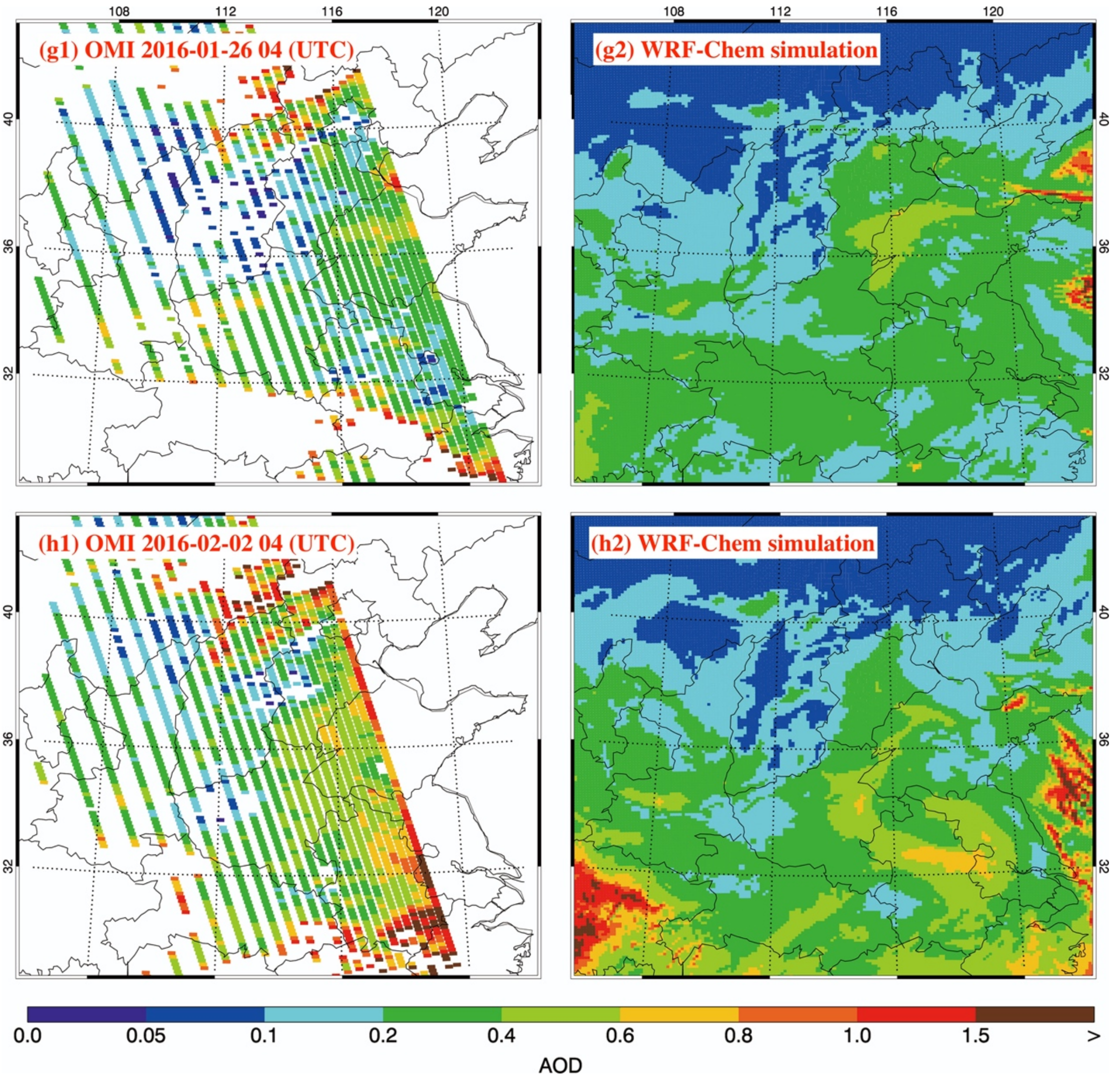

Fig. S17 Continued.

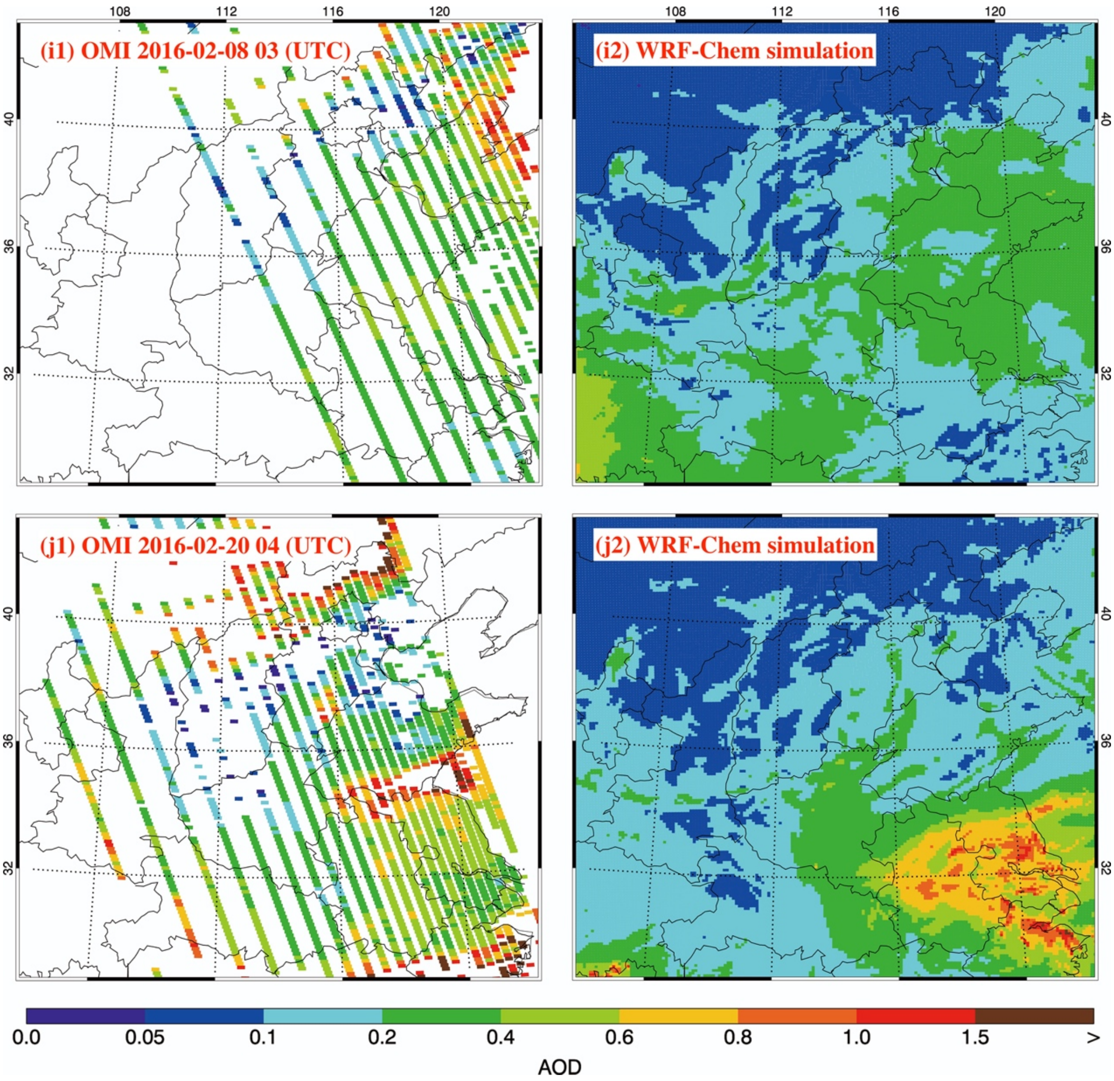

Fig. S17 Continued.

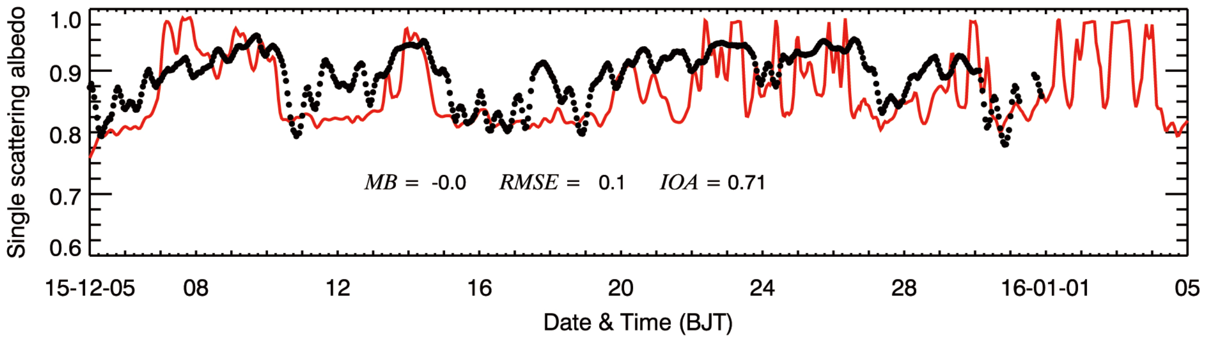

Fig. S18. Comparison of measured (black dots) and predicted (red line) diurnal profiles of SSA at 520nm in Beijing from 05 December 2015 to 04 January 2016.

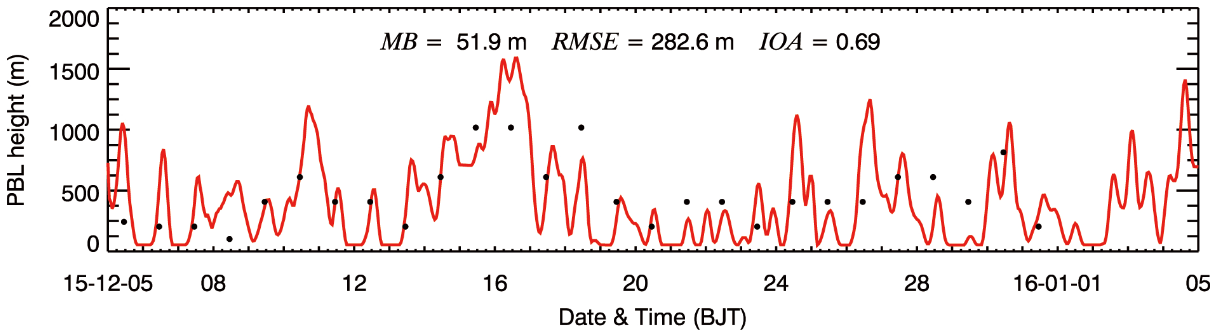

Fig. S19. Comparison of predicted diurnal profile (red line) of PBLH from 05 December 2015 to 04 January 2016 with observations at 12:00 BJT in Beijing.

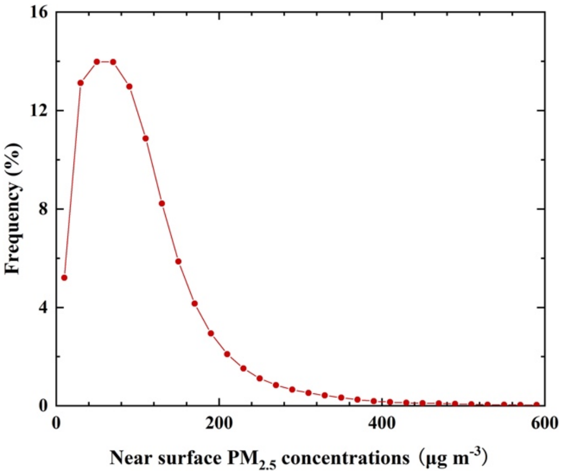

Fig. S20. Occurrence frequency of different $PM_{2.5}$ bins in the NCP from 05 December 2015 to 06 March 2016.

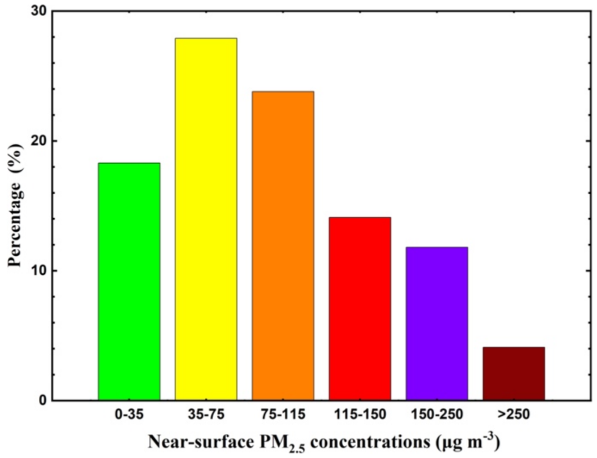

Fig. S21. Occurrence frequency of different polluted level in the NCP from 05 December 2015 to 06 March 2016.

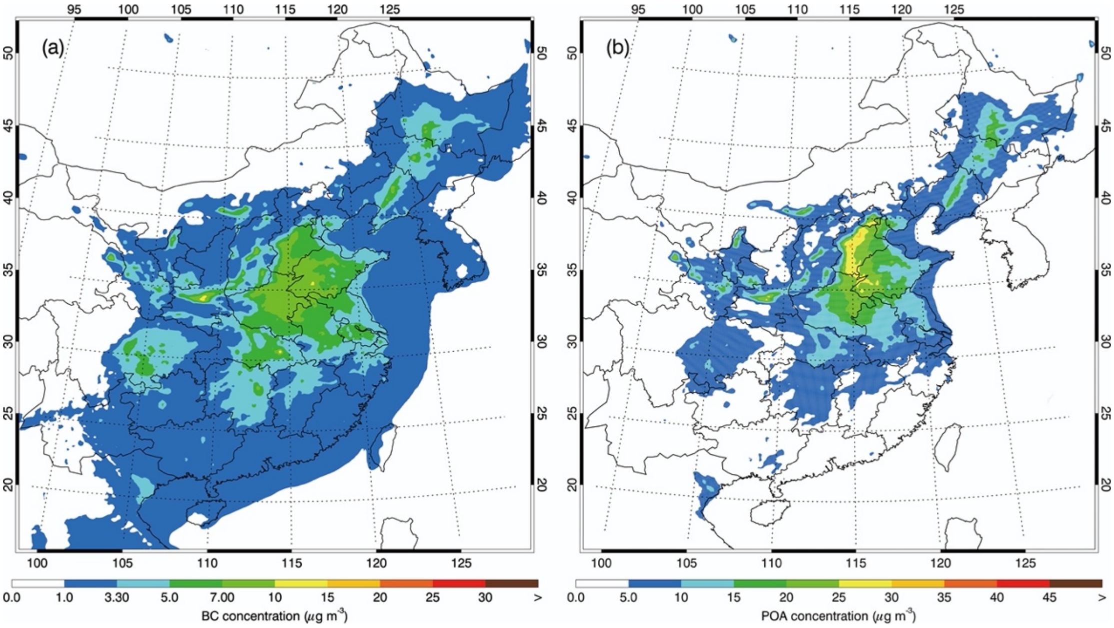

Fig. S22. Spatial distribution of average (a) BC and (b) POA concentrations in F_{BASE} from 05 December 2015 to 06 March 2016.

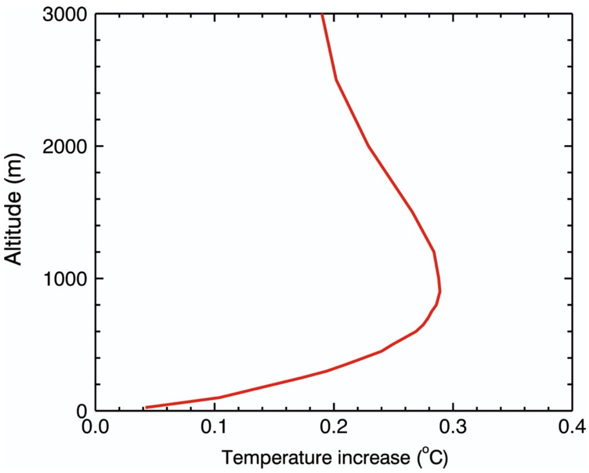

Fig. S23. Variations of the average temperature increase caused by AARI with altitude in the NCP from 05 December 2015 to 06 March 2016.

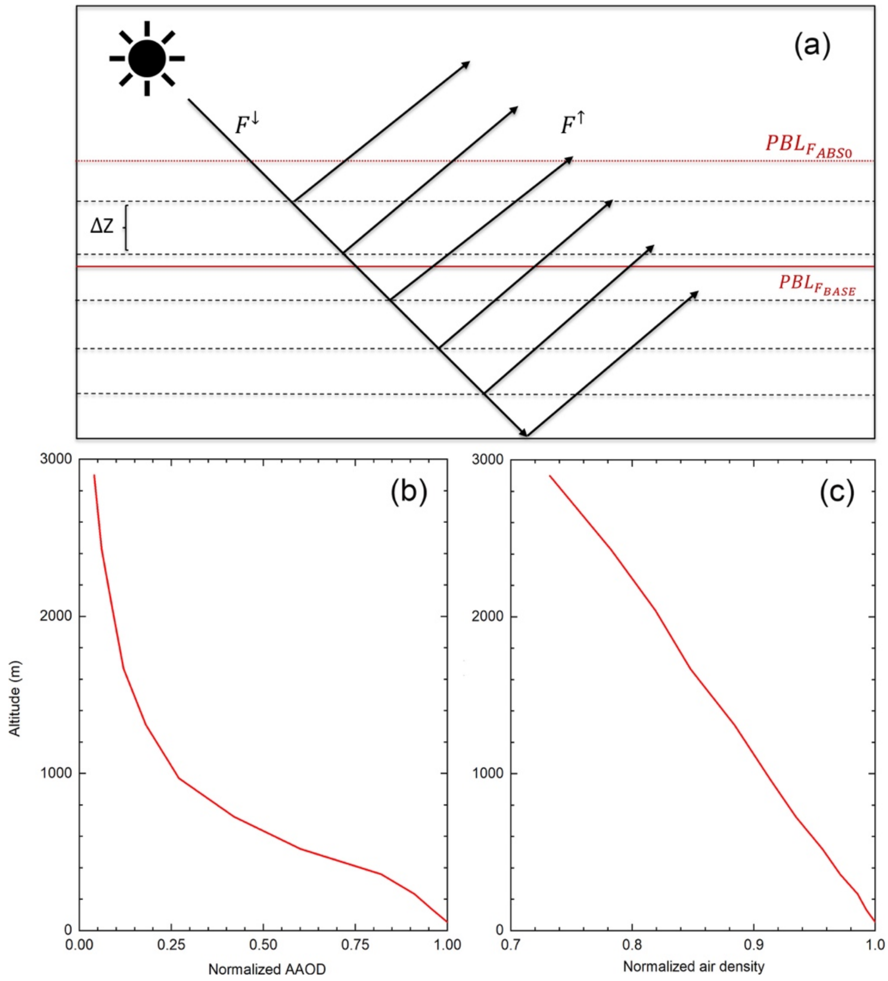

Fig. S24. (a) Upward and downward solar flux absorbed by the light absorbing aerosols; average vertical profile of (b) normalized AAOD and (c) normalized air density with altitude in the NCP from 05 December 2015 to 06 March 2016.

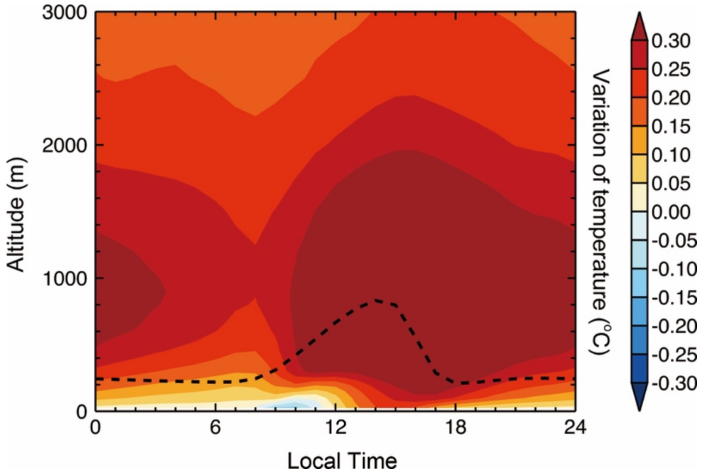

Fig. S25. Daily evolution of vertical distribution of the temperature variation due to AARI in the NCP with hourly $PM_{2.5}$ concentrations higher than 75 μg m^{-3} from 05 December 2015 to 06 March 2016. The dashed line in (b) represents the PBL height in F_{BASE}.

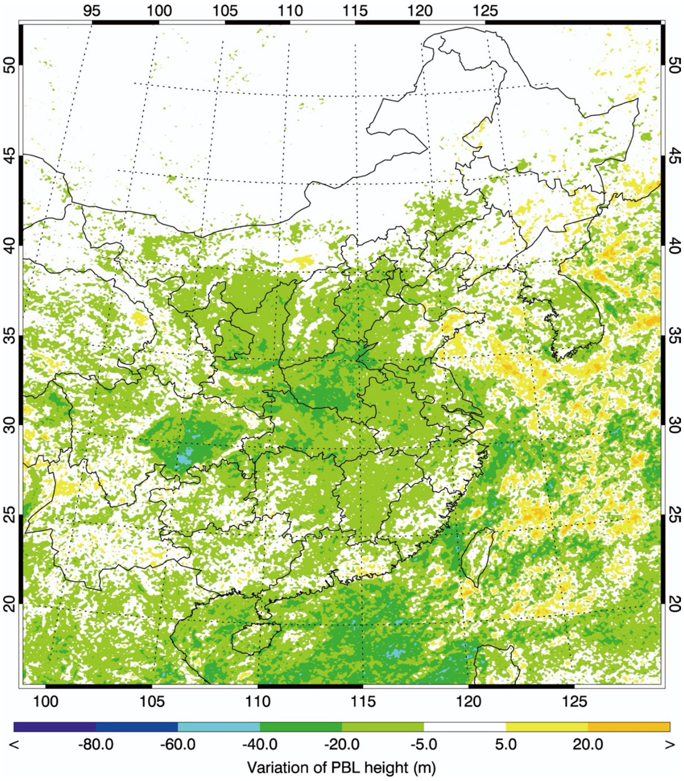

Fig. S26. Variation of average daytime PBLH caused by AARI from 05 December 2015 to 06 March 2016.

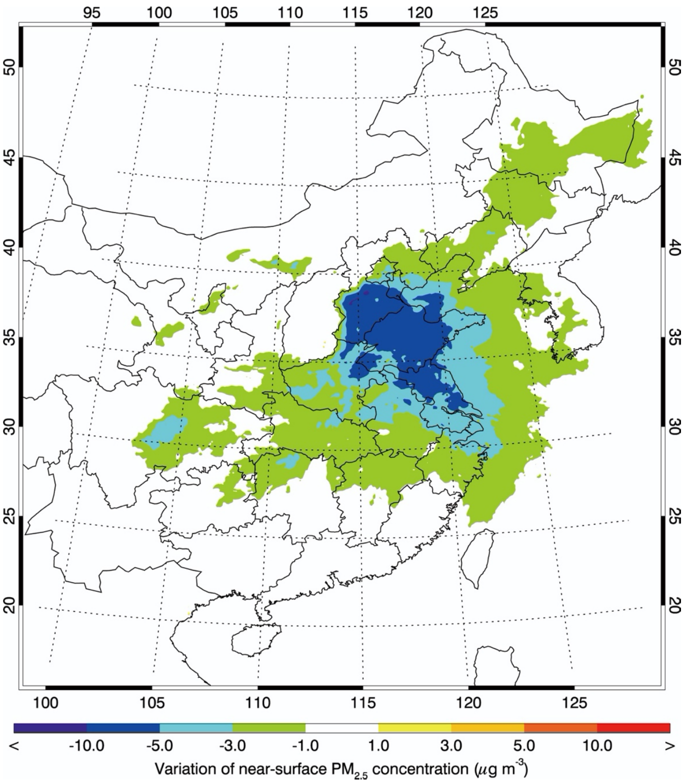

Fig. S27. Spatial distribution of the variation of average near-surface [$PM_{2.5}$] caused by AARI from 05 December 2015 to 06 March 2016.

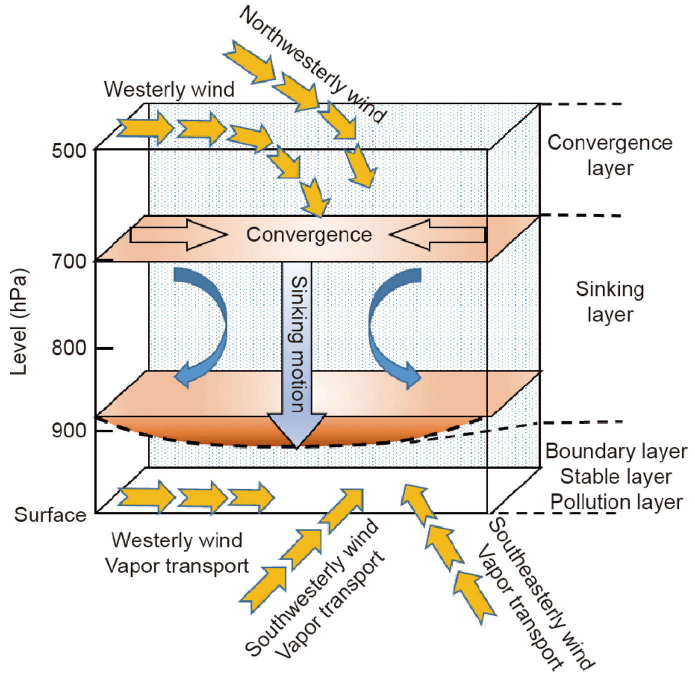

Fig. S28. A schematic of dynamic conditions for the formation of a severe persistent haze event in North China. Adapted from Wu et al. (*47*).

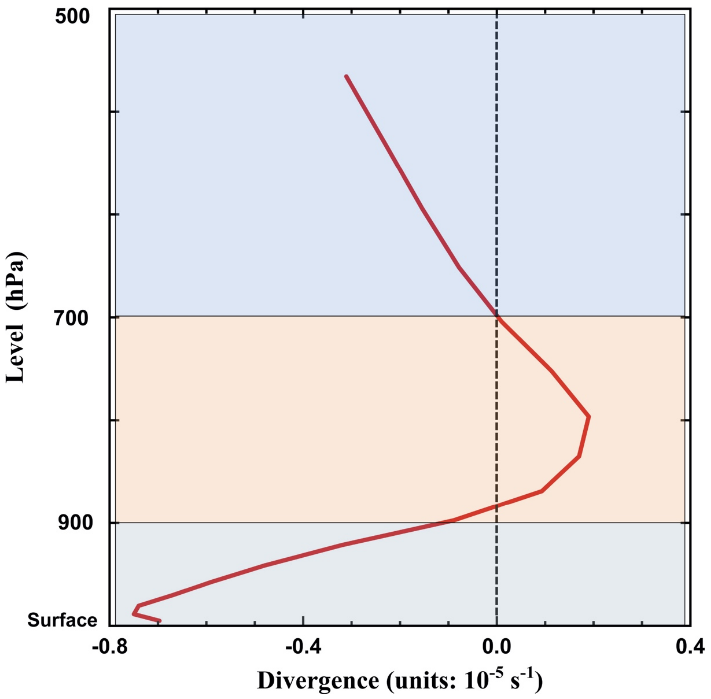

Fig. S29. Vertical distribution of wind divergence in the NCP from 05 December 2015 to 06 March 2016.

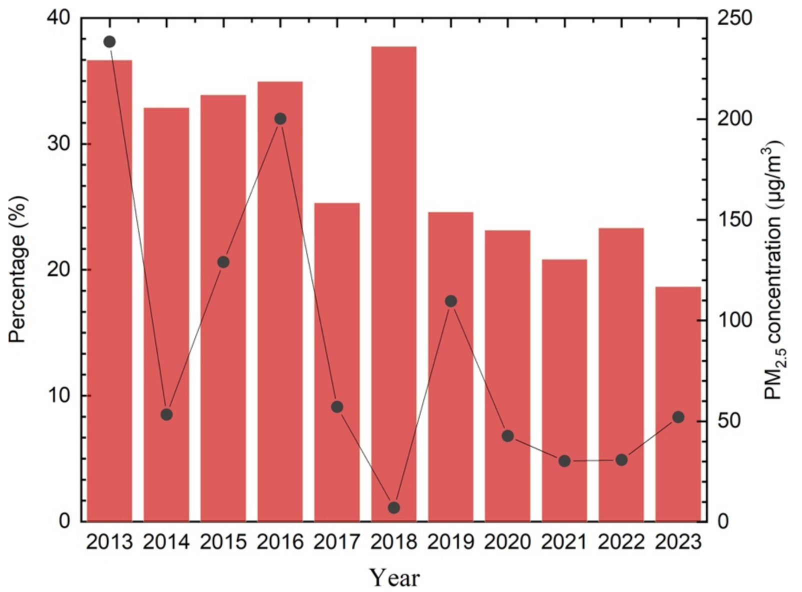

Fig. S30. Proportion of polluted days (hourly $PM_{2.5}$ concentration > 75 μg m^{-3} and lasing two or more days) with average $PM_{2.5}$ concentration during the wintertime from 2013 to 2023.

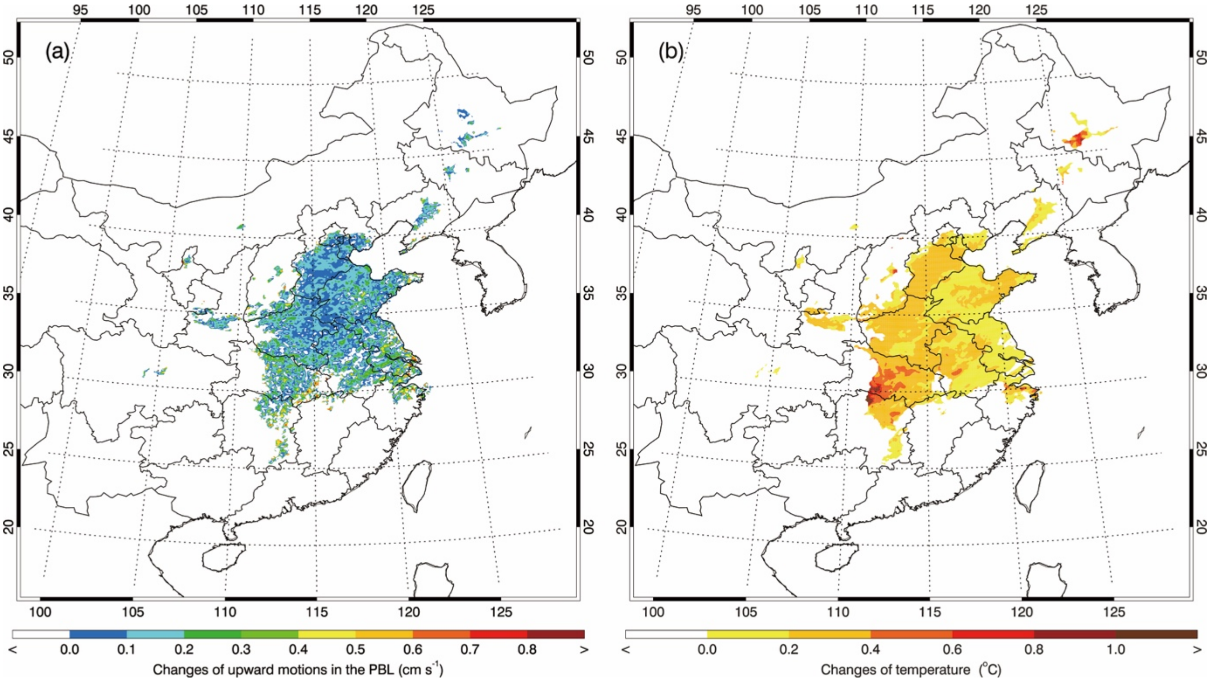

Fig. S31. Spatial distribution of (a) changes of upward motions in the PBL and (b) changes of temperature at around 900m during the polluted episode when heavy PM pollution (near-surface [$PM_{2.5}$] more than 250 μg m^{-3}) occurs from 05 December 2015 to 06 March 2016.

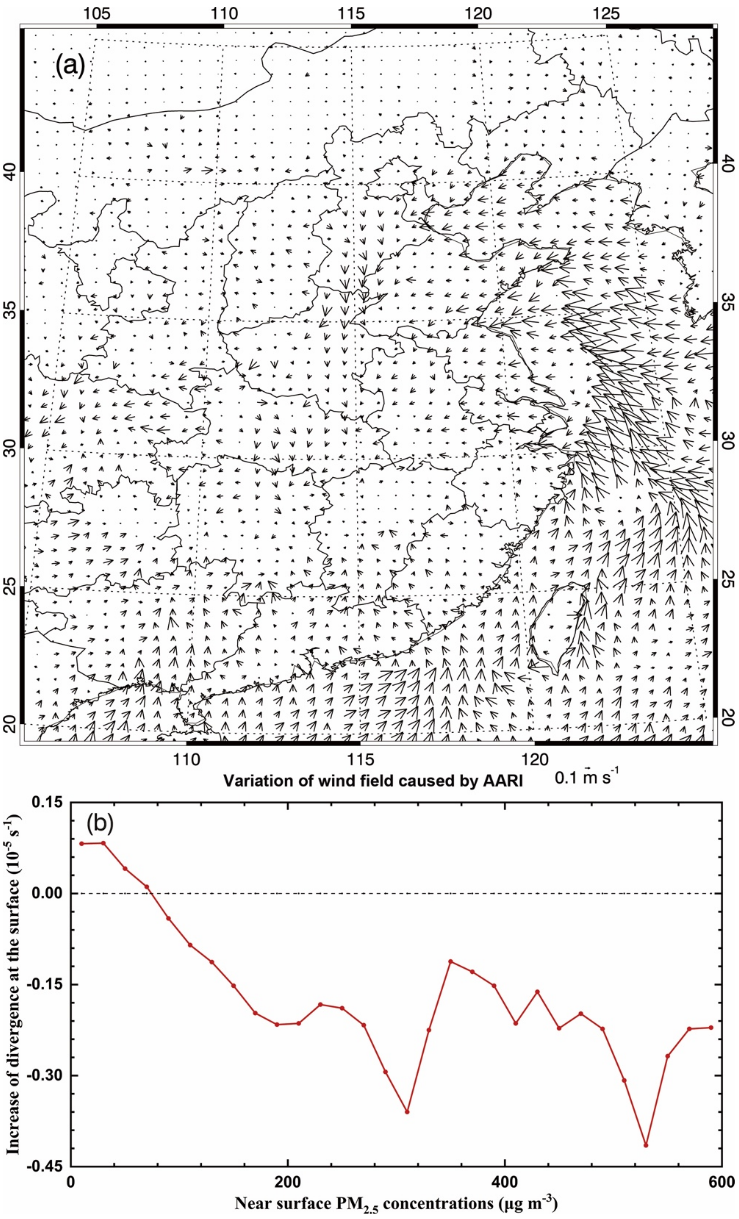

Fig. S32. (a) Average variation of near-surface wind fields caused by AARI, and (b) average increase of divergence at the surface as a function of near-surface [$PM_{2.5}$] in the NCP from 05 December 2015 to 06 March 2016.

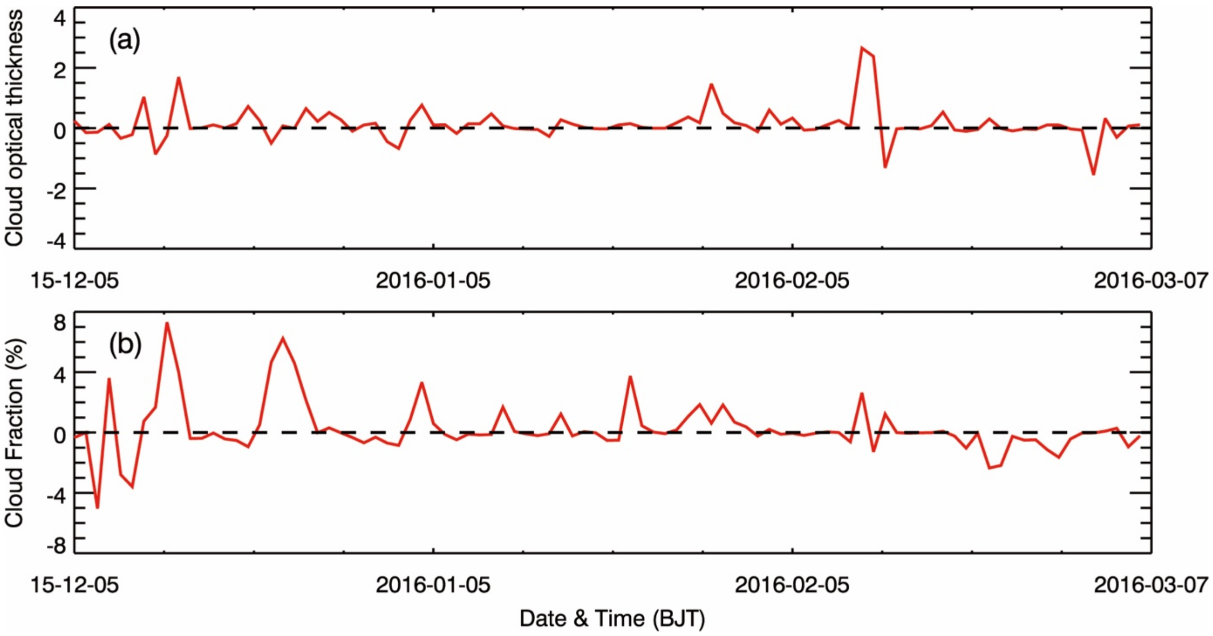

Fig. S33. Daily profiles of variation of average COT and CF caused by AARI in the NCP from 05 December 2015 to 06 March 2016.

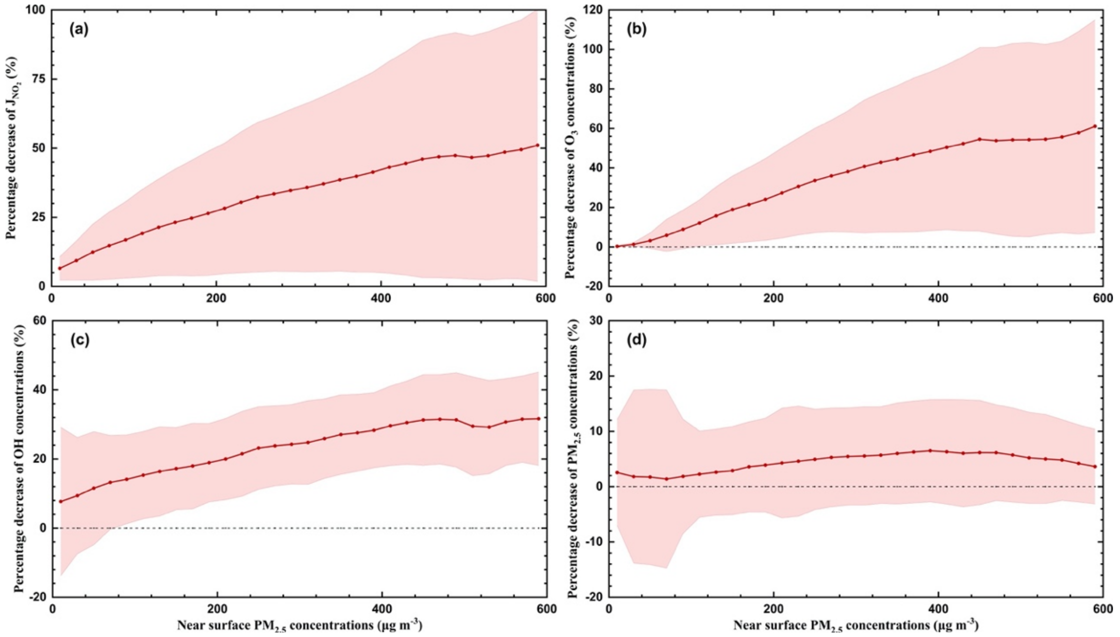

Fig. S34. Percentage decrease of daytime (a) $\boldsymbol{J_{NO_2}}$, (b) O_3 concentrations, (c) OH concentrations and (d) the all-day [$PM_{2.5}$] caused by AAPI with error shadow as a function of near-surface [$PM_{2.5}$] in the NCP from 05 December 2015 to 06 March 2016.

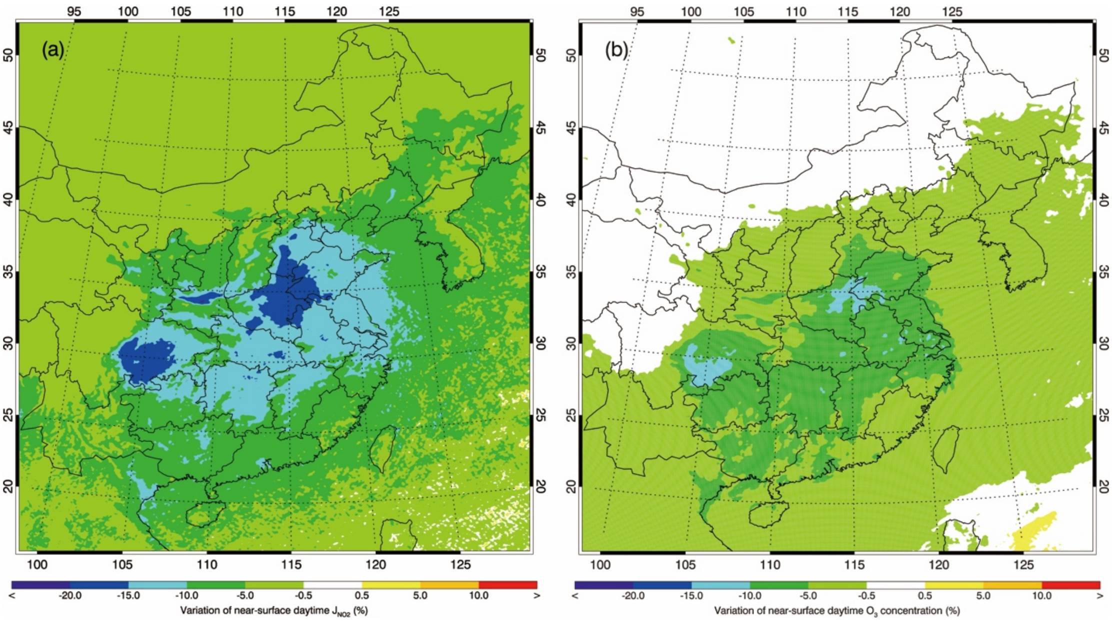

Fig. S35. Spatial distribution of the percentage variation of average daytime $\boldsymbol{J_{NO_2}}$ and O_3 concentrations caused by AAPI from 05 December 2015 to 06 March 2016.

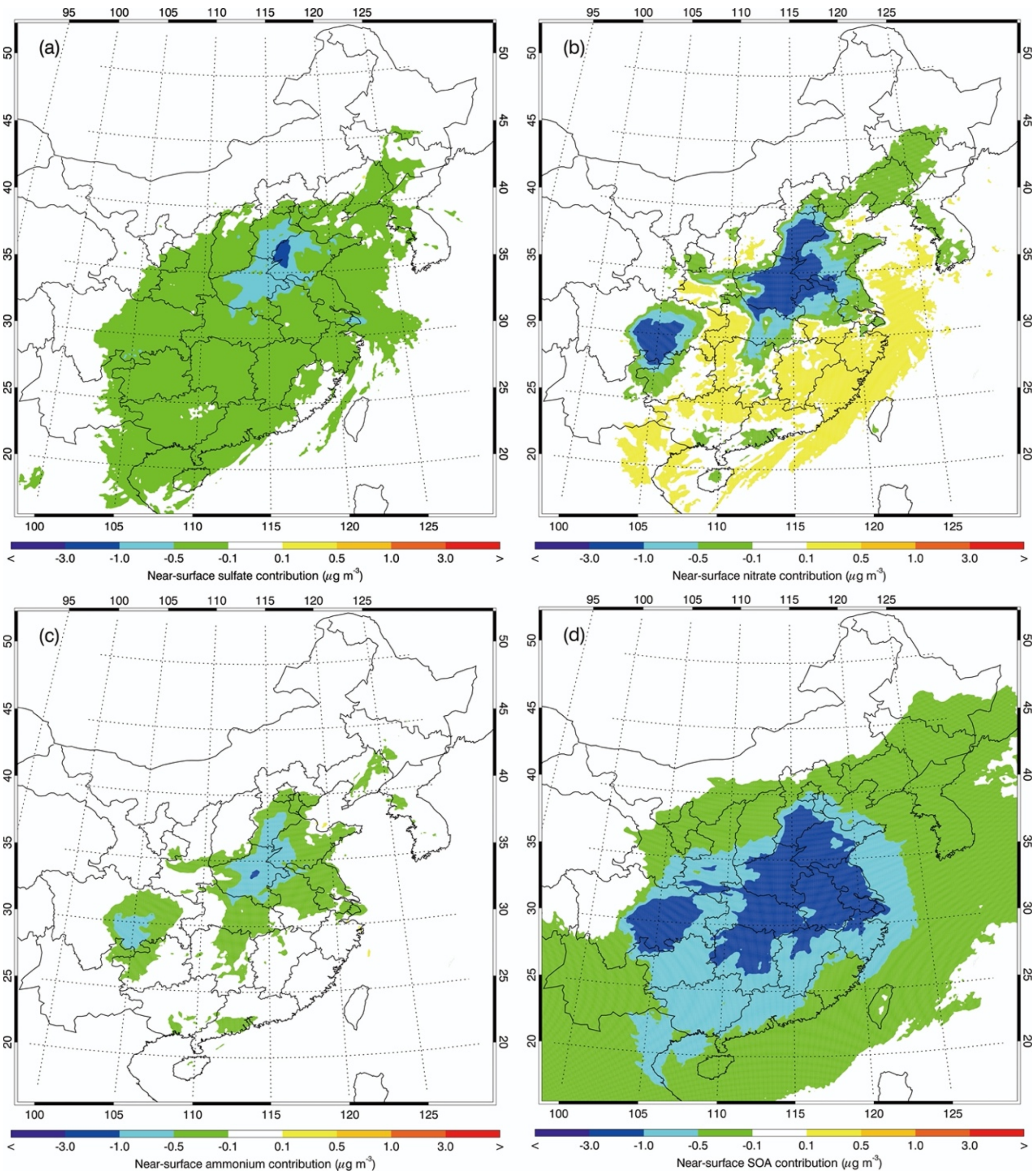

Fig. S36. Spatial distribution of the variation of average near-surface (a) sulfate, (b) nitrate, (c) ammonium, and (d) SOA concentrations due to AAPI from 05 December 2015 to 06 March 2016.

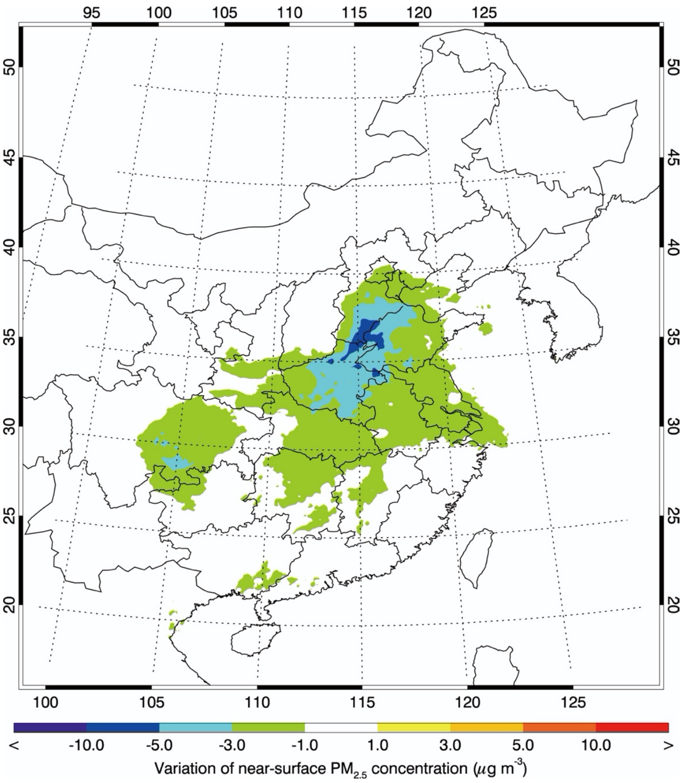

Fig. S37. Spatial distribution of the variation of average near-surface [$PM_{2.5}$] caused by AAPI from 05 December 2015 to 06 March 2016.

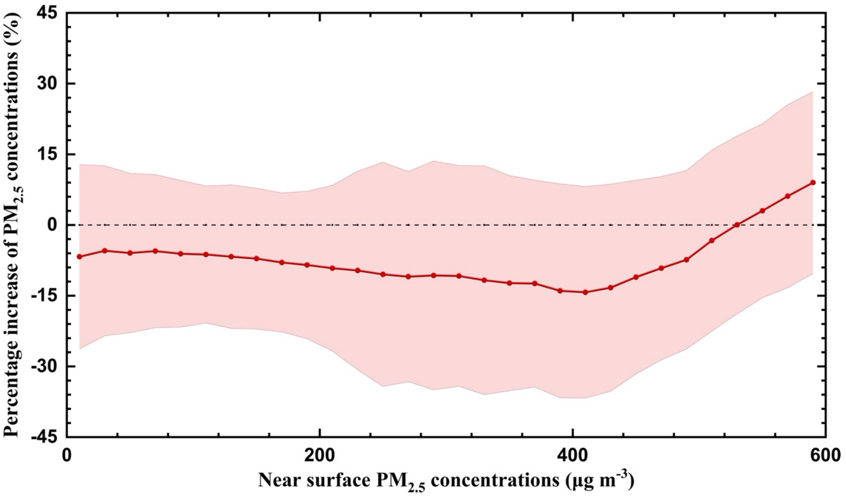

Fig. S38. Percentage increase of near-surface [$PM_{2.5}$] caused by the synergistic effect of AARI and AAPI with error shadow as a function of [$PM_{2.5}$] in the NCP from 05 December 2015 to 06 March 2016.

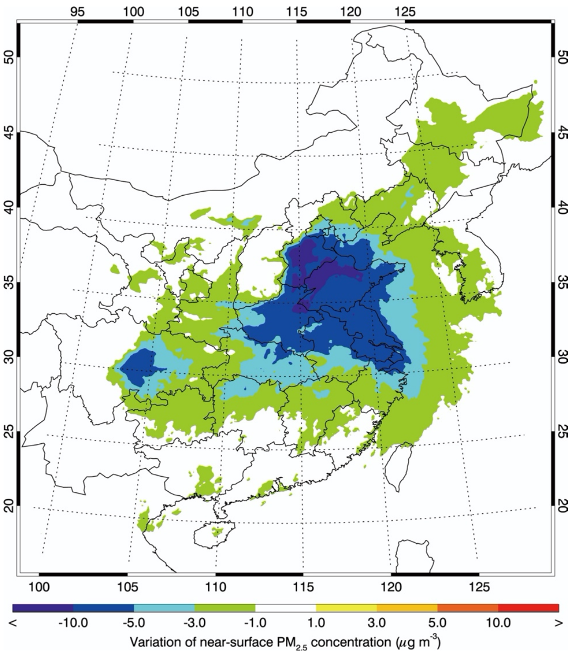

Fig. S39. Spatial distribution of the variation of average near-surface [$PM_{2.5}$] caused by the synergistic effect of AARI and AAPI from 05 December 2015 to 06 March 2016.

SI References

1. G. A. Grell *et al.*, Fully coupled "online" chemistry within the WRF model. *Atmospheric Environment* 39, 6957-6975 (2005).
2. G. Li *et al.*, Impacts of HONO sources on the photochemistry in Mexico City during the MCMA-2006/MILAGO Campaign. *Atmospheric Chemistry and Physics* 10, 6551-6567 (2010).
3. G. Li, N. Bei, X. Tie, L. T. Molina, Aerosol effects on the photochemistry in Mexico City during MCMA-2006/MILAGRO campaign. *Atmospheric Chemistry and Physics* 11, 5169-5182 (2011).
4. G. Li *et al.*, Simulations of organic aerosol concentrations in Mexico City using the WRF-CHEM model during the MCMA-2006/MILAGRO campaign. *Atmospheric Chemistry and Physics* 11, 3789-3809 (2011).
5. F. S. Binkowski, S. J. Roselle, Models-3 community multiscale air quality (CMAQ) model aerosol component - 1. Model description. *Journal of Geophysical Research-Atmospheres* 108, 18 (2003).
6. M. Kulmala, A. Laaksonen, L. Pirjola, Parameterizations for sulfuric acid/water nucleation rates. *Journal of Geophysical Research Atmospheres* 103, 8301-8308 (1998).
7. M. L. Wesely, Parameterization of surface resistances to gaseous dry deposition in regional-scale numerical models. *Atmospheric Environment* 23, 1293-1304 (1989).
8. Li, R. Y. Zhang, J. W. Fan, X. X. Tie, Impacts of black carbon aerosol on photolysis and ozone. *Journal of Geophysical Research-Atmospheres* 110, 10 (2005).
9. A. Nenes, S. N. Pandis, C. Pilinis, ISORROPIA: A new thermodynamic equilibrium model for multiphase multicomponent inorganic aerosols. *Aquatic Geochemistry.* 4, 123-152 (1998).
10. M. K. Shrivastava, T. E. Lane, N. M. Donahue, S. N. Pandis, A. L. Robinson, Effects of gas particle partitioning and aging of primary emissions on urban and regional organic aerosol concentrations. *Journal of Geophysical Research-Atmospheres* 113, 16 (2008).
11. A. L. Robinson *et al.*, Rethinking organic aerosols: Semivolatile emissions and photochemical aging. *Science* 315, 1259-1262 (2007).
12. J. Liggio, S. M. Li, R. McLaren, Reactive uptake of glyoxal by particulate matter. *Journal of Geophysical Research-Atmospheres* 110, 13 (2005).
13. J. Zhao, N. P. Levitt, R. Y. Zhang, J. M. Chen, Heterogeneous reactions of

methylglyoxal in acidic media: Implications for secondary organic aerosol formation. *Environmental Science and Technology* 40, 7682-7687 (2006).

14. R. Volkamer *et al.*, A missing sink for gas-phase glyoxal in Mexico City: Formation of secondary organic aerosol. *Geophysical Research Letters* 34, 5 (2007).

15. S.-Y. Hong, J.-O. J. Lim, The WRF Single-Moment 6-Class Microphysics Scheme (WSM6). *Asia-Pacific Journal of Atmospheric Sciences* 42, 129-151 (2006).

16. Z. I. Janjić, Nonsingular Implementation of the Mellor–Yamada Level 2.5 Scheme in the NCEP Meso Model. *Ncep Office Note* 436, (2002).

17. F. Chen, J. Dudhia, Coupling an advanced land surface-hydrology model with the Penn State-NCAR MM5 modeling system. Part I: Model implementation and sensitivity. *Monthly Weather Review* 129, 569-585 (2001).

18. M. D. Chou, M. J. Suarez, X. Z. Liang, M. H. Yan, C. Cote, A Thermal Infrared Radiation Parameterization for Atmospheric Studies. *Max J*, (2001).

19. M. D. Chou, M. J. Suarez, A solar radiation parameterization for atmospheric studies, NASA TM-104606. *Nasa Tech.memo* 15, (1999).

20. L. W. Horowitz *et al.*, A global simulation of tropospheric ozone and related tracers: Description and evaluation of MOZART, version 2. *Journal of Geophysical Research-Atmospheres* 108, 29 (2003).

21. Q. Zhang *et al.*, Asian emissions in 2006 for the NASA INTEX-B mission. *Atmospheric Chemistry and Physics* 9, 5131-5153 (2009).

22. G. H. Li *et al.*, Widespread and persistent ozone pollution in eastern China during the non-winter season of 2015: observations and source attributions. *Atmospheric Chemistry and Physics* 17, 2759-2774 (2017).

23. A. Guenther *et al.*, Estimates of global terrestrial isoprene emissions using MEGAN (Model of Emissions of Gases and Aerosols from Nature). *Atmospheric Chemistry and Physics* 6, 3181-3210 (2006).

24. C. Wiedinmyer *et al.*, Estimating emissions from fires in North America for air quality modeling. *Atmospheric Environment* 40, 3419-3432 (2006).

25. C. Wiedinmyer *et al.*, The Fire INventory from NCAR (FINN): a high resolution global model to estimate the emissions from open burning. *Geoscientific Model Development* 4, 625-641 (2011).

26. G. A. D'Almeida, P. Koepke, E. P. Shettle, Atmospheric aerosols: Global climatology and radiative characteristics. *Journal of Medical Microbiology* 54, 55-61 (1991).

27. R. C. Moffet, K. A. Prather, In-situ measurements of the mixing state and optical properties of soot with implications for radiative forcing estimates. *Proceedings of the National Academy of Sciences of the United States of America* 106, 11872-11877 (2009).
28. W. F. Cooke, J. J. N. Wilson, A global black carbon aerosol model. *Journal of Geophysical Research-Atmospheres* 101, 19395-19409 (1996).
29. J. C. Barnard, R. Volkamer, E. I. Kassianov, Estimation of the mass absorption cross section of the organic carbon component of aerosols in the Mexico City Metropolitan Area. *Atmospheric Chemistry and Physics* 8, 6665-6679 (2008).
30. T. W. Kirchstetter, T. Novakov, P. V. Hobbs, Evidence that the spectral dependence of light absorption by aerosols is affected by organic carbon. *Journal of Geophysical Research-Atmospheres* 109, 12 (2004).
31. Q. L. Wang *et al.*, Vertical evolution of black and brown carbon during pollution events over North China Plain. *Science of the Total Environment* 806, (2022).
32. H. Liu, B. Hu, L. Zhang, Y. S. Wang, P. F. Tian, Spatiotemporal characteristics of ultraviolet radiation in recent 54years from measurements and reconstructions over the Tibetan Plateau. *Journal of Geophysical Research-Atmospheres* 121, 7673-7690 (2016).
33. N. F. Bei *et al.*, Impacts of meteorological uncertainties on the haze formation in Beijing-Tianjin-Hebei (BTH) during wintertime: a case study. *Atmospheric Chemistry and Physics* 17, 14579-14591 (2017).
34. X. Li *et al.*, Contributions of residential coal combustion to the air quality in Beijing-Tianjin-Hebei (BTH), China: a case study. *Atmospheric Chemistry and Physics* 18, 10675-10691 (2018).
35. N. Bei, W. Lei, M. Zavala, L. T. Molina, Ozone predictabilities due to meteorological uncertainties in the Mexico City basin using ensemble forecasts. *Atmospheric Chemistry and Physics* 10, 6295-6309 (2010).
36. Bei, G. Li, L. T. Molina, Uncertainties in SOA simulations due to meteorological uncertainties in Mexico City during MILAGRO-2006 field campaign. *Atmospheric Chemistry and Physics* 12, 11295-11308 (2012).
37. G. H. Li *et al.*, A possible pathway for rapid growth of sulfate during haze days in China. *Atmospheric Chemistry and Physics* 17, 3301-3316 (2017).
38. G. Wang *et al.*, Persistent sulfate formation from London Fog to Chinese haze. *Proceedings of the National Academy of Sciences of the United States of America* 48, 13630-13635 (2016).

39. Y. F. Cheng *et al.*, Reactive nitrogen chemistry in aerosol water as a source of sulfate during haze events in China. *Science Advances* 2, 11 (2016).

40. A. Engstrom, A. M. L. Ekman, Impact of meteorological factors on the correlation between aerosol optical depth and cloud fraction. *Geophysical Research Letters* 37, 4 (2010).

41. D. Chand *et al.*, Aerosol optical depth increase in partly cloudy conditions. *Journal of Geophysical Research-Atmospheres* 117, 8 (2012).

42. B. S. Grandey, P. Stier, T. M. Wagner, Investigating relationships between aerosol optical depth and cloud fraction using satellite, aerosol reanalysis and general circulation model data. *Atmospheric Chemistry and Physics* 13, 3177-3184 (2013).

43. S. K. Satheesh, V. Vinoj, K. Krishnamoorthy, Assessment of Aerosol Radiative Impact over Oceanic Regions Adjacent to Indian Subcontinent Using Multisatellite Analysis. *Advances in Meteorology* 13 (2010).

44. C. Sivaraman, McFarlane, S., and Chapman E., Planetary bound- ary layer (PBL) height value added product (VAP): Radiosonde retrievals [J/OL]. *U.S. DOE, Office of Science, Office of Biological and Environment Research, DOE/SC-ARM/TR-132*, (2013).

45. J. W. Fan, R. Y. Zhang, W. K. Tao, K. I. Mohr, Effects of aerosol optical properties on deep convective clouds and radiative forcing. *Journal of Geophysical Research-Atmospheres* 113, (2008).

46. T. C. Bond *et al.*, Bounding the role of black carbon in the climate system: A scientific assessment. *Journal of Geophysical Research-Atmospheres* 118, 5380-5552 (2013).

47. P. Wu, Y. H. Ding, Y. J. Liu, Atmospheric circulation and dynamic mechanism for persistent haze events in the Beijing-Tianjin-Hebei region. *Advances in Atmospheric Sciences* 34, 429-440 (2017).
